# Non-equilibrium processing leads to record high thermoelectric figure of merit in PbTe–SrTe

**DOI:** 10.1038/ncomms12167

**Published:** 2016-07-26

**Authors:** Gangjian Tan, Fengyuan Shi, Shiqiang Hao, Li-Dong Zhao, Hang Chi, Xiaomi Zhang, Ctirad Uher, Chris Wolverton, Vinayak P. Dravid, Mercouri G. Kanatzidis

**Affiliations:** 1 Department of Chemistry, Northwestern University, Evanston, Illinois 60208, USA; 2 Department of Materials Science and Engineering, Northwestern University, Evanston, Illinois 60208, USA; 3 School of Materials Science and Engineering, Beihang University, Beijing 100191, China; 4 Department of Physics, University of Michigan, Ann Arbor, Michigan 48109, USA; 5 Materials Science Division, Argonne National Laboratory, Argonne, Illinois 60439, USA

## Abstract

The broad-based implementation of thermoelectric materials in converting heat to electricity hinges on the achievement of high conversion efficiency. Here we demonstrate a thermoelectric figure of merit *ZT* of 2.5 at 923 K by the cumulative integration of several performance-enhancing concepts in a single material system. Using non-equilibrium processing we show that hole-doped samples of PbTe can be heavily alloyed with SrTe well beyond its thermodynamic solubility limit of <1 mol%. The much higher levels of Sr alloyed into the PbTe matrix widen the bandgap and create convergence of the two valence bands of PbTe, greatly boosting the power factors with maximal values over 30 μW cm^−1^ K^−2^. Exceeding the 5 mol% solubility limit leads to endotaxial SrTe nanostructures which produce extremely low lattice thermal conductivity of 0.5 W m^−1^ K^−1^ but preserve high hole mobilities because of the matrix/precipitate valence band alignment. The best composition is hole-doped PbTe–8%SrTe.

## Abstract (web summary)

Alloying lead telluride has proven a good strategy to improve its thermoelectric properties. Here, the authors use a rapid temperature quenching to prepare compounds alloyed above the thermodynamic solubility limit, which results in band convergence and high thermoelectric figures of merit.

## Graphical abstract

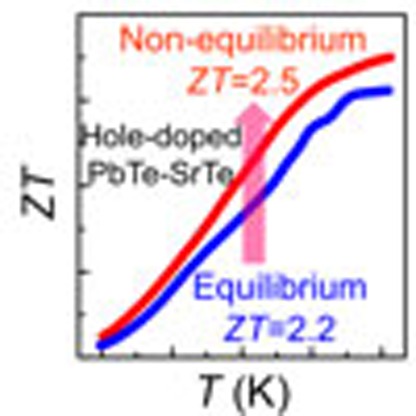

Thermoelectric materials technology can play a role in energy-saving alternatives for sustainable power generation, by providing a direct means for heat to electricity conversion^1^,^2^,^3^,^4^,^5^,^6^. The efficiency of a thermoelectric material is defined by the figure of merit *ZT*=*S*^2^*σT*/*κ*=*S*^2^*σT*/(*κ*_el_+*κ*_lat_), where *S* is the Seebeck coefficient, *σ* is the electrical conductivity, *T* is the temperature and *κ* is the thermal conductivity that is comprised of an electronic part (*κ*_el_) and a lattice part (*κ*_lat_). PbTe-based materials are the top-performing thermoelectrics in the temperature range of 500–900 K (refs 7, 8, 9, 10, 11), thanks to a unique two-valence-band structure: a primary light hole band at the L point (L band) and a second lower-lying heavy hole band at the Σ point (Σ band) with an energy separation (Δ*E*_L−Σ_) of 0.15–0.20 eV at 300 K between them^12^,^13^. Recent studies^14^,^15^,^16^ show that Mg or Mn substitution in PbTe can decrease Δ*E*_L−Σ_ to within a few *k*_B_*T* (*k*_B_ is the Boltzmann constant). This decrease is referred to as effective band convergence, which makes the contribution of the Σ band to the charge transport very significant, especially when temperature is higher than 500 K, and produces much higher Seebeck coefficients^9^,^17^.

In addition to band structure engineering, nanostructuring is an efficient route to enhancing the *ZT* of PbTe by strongly reducing the thermal conductivity. For instance, previous studies showed only a small equilibrium solid state solubility (less than1 mol%) of SrTe in bulk PbTe (refs 7, 8). Samples prepared under thermodynamic control, using slow cooling procedures of molten mixtures (11 K h^−1^ from 1,323 to 873 K (ref. 7), dissolve only very small amounts of SrTe. Any excess SrTe emerges as strained endotaxial nanostructures whose valence band is almost aligned with that of PbTe. This matrix/precipitate valence band alignment mitigates the scattering of holes, while the added interfaces created by SrTe nanostructuring strongly impede phonon propagation^7^. When coupled with the atomic scale point defects created by Na doping and mesoscale structuring using spark plasma sintering (SPS), these combined effects can lead to a *ZT* of ∼2.2 at 913 K (ref. 8).

Although the thermodynamic solubility limit of SrTe in PbTe is low (<1 mol%), early studies of PbTe–SrTe thin films^18^,^19^ grown under kinetic control using molecular beam epitaxy showed that up to 15 mol% of SrTe can be dissolved into PbTe. If this higher SrTe alloying fraction can be realized in bulk PbTe, our calculations predict it would lead to valence band convergence, an effect which is negligible in the low solubility samples^7^,^8^. It should be noted that in previous studies on PbTe–SrTe thin films^19^, no such a valence band convergence effect was observed because the thin films were n-type doped. The theoretical prediction motivated us to pursue kinetically controlled methods for fabricating PbTe samples heavily alloyed with SrTe. Herein, we show that via a non-equilibrium route that involves rapid ice water quenching (estimated cooling rate higher than 100 K/s, based on the time it took before the melt was completely cooled to room temperature, which enables the fine element distribution) of the 1,423 K melt followed by annealing at 873 K (which serves as kinetic control) we can produce hole-doped samples that achieve a 5 mol% solubility of SrTe in bulk PbTe. Exceeding the 5 mol% limit by using excess SrTe then leads to ubiquitous nanostructures.

We show here that the high degree of SrTe alloying produces a material that simultaneously incorporates four practical performance-enhancing mechanisms: valence band convergence of PbTe, a strengthened point defect phonon scattering when SrTe is alloyed, and extensive nanostructuring with good valence band alignment of precipitate/matrix when excess SrTe is added. The result is a highly optimized material with a high *ZT* of ∼2.5 at 923 K coupled with a new milestone of average *ZT* of 1.67 between 300 and 900 K in Na-doped PbTe–8%SrTe. This new approach is quite different from previous studies on PbTe (for example PbTe_0.7_S_0.3_ and (PbTe)_0.65_(PbS)_0.25_(PbSe)_0.1_ (refs 20, 21) where only a few of the above mechanisms are combined. We hope this approach can be equally applied to other thermoelectric materials for improved performance.

## Results

### SrTe alloying in PbTe and electronic structure modification

**Figure 1:**
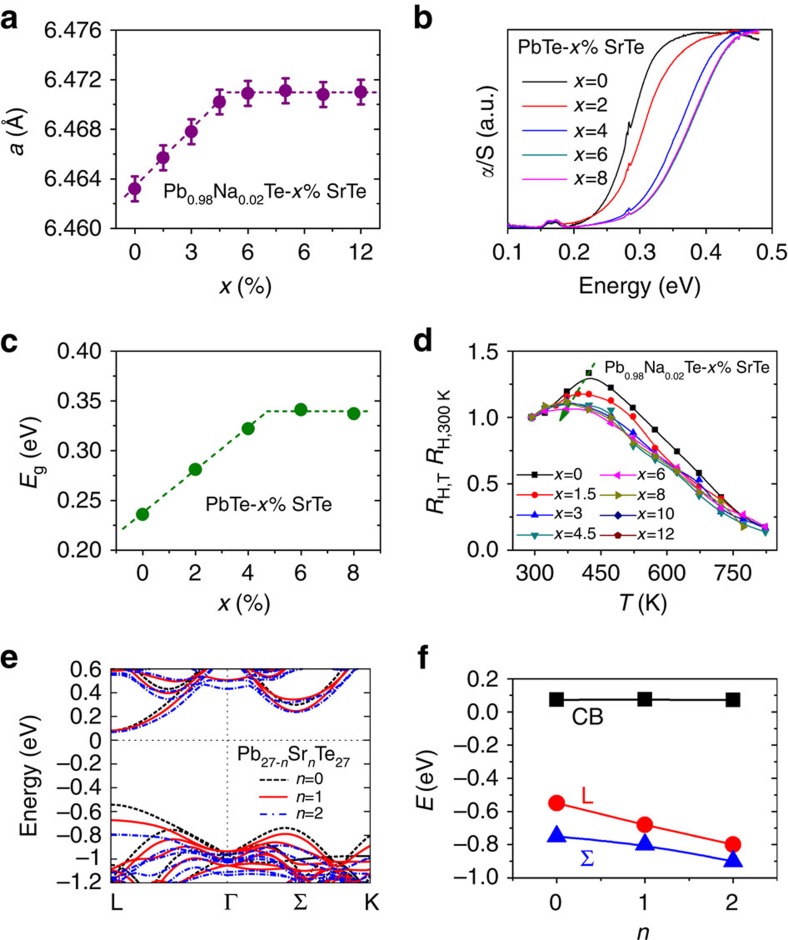
Heavy SrTe alloying in PbTe and its impact on the band structure. (**a**) Lattice parameter (*a*) as a function of SrTe content in samples of Pb_0.98_Na_0.02_Te–*x*%SrTe prepared by non-equilibrium processing. 0.001 Å error bars are applied. The dotted lines are fitting of *a* on the basis of Vegard's law, where the cross-point coordinate (4.91, 6.47) suggests a solubility limit of 5 mol% for SrTe in PbTe. (**b**) Infrared absorption spectra for PbTe–*x*%SrTe samples without Na doping. (**c**) Bandgap enlargement of PbTe with increasing SrTe fraction. The cross-point coordinate (4.68, 0.34) of the two fitting lines indicates that the bandgap enlargement saturates at *x*=5. (**d**) Temperature dependence of Hall coefficients (*R*_H_, normalized to room temperature values) for Pb_0.98_Na_0.02_Te–*x*%SrTe. (**e**) First-principles band structure calculations of Pb_27−*n*_Sr_*n*_Te_27_ (*n*=0, 1 and 2), where spin–orbit interaction is not considered. The black, red and blue lines represent the theoretical band structures for *n*=0, 1 and 2, respectively. (**f**) The energy variations of the conduction band (CB), primary valence band (L) and second valence band (Σ) as a function of *n*.

Within the detection limit of laboratory X-ray diffraction, all the Pb_0.98_Na_0.02_Te–*x*%SrTe samples in this study, prepared under non-equilibrium conditions, are single phase compounds crystallizing in the rock salt structure (Supplementary Fig. 1). The lattice parameters shown in Fig. 1a increase almost linearly with increasing SrTe content up to *x*=6 and beyond that remain unchanged. The lattice expansion of PbTe upon SrTe addition is consistent with the larger radius of Sr^2+^ (1.32 Å) in comparison to Pb^2+^ (1.19 Å) and confirms the successful substitution of Sr for Pb in PbTe. We also note that water quenching produces a behaviour of the lattice parameter (Supplementary Fig. 2) and band structure (Supplementary Fig. 3) similar to that obtained by molecular beam epitaxy^18^,^19^, which further supports the effectiveness of non-equilibrium synthesis in producing a high degree alloying fraction of SrTe in PbTe. By fitting the lattice parameter as a function of SrTe content (on the basis of Vegard's law) one can conclude that the achieved solubility limit of SrTe in these samples is around 5 mol%. This value is significantly higher than the previously achieved <1 mol% under equilibrium synthesis conditions^7^,^8^.

Infrared absorption spectra for low carrier density PbTe–*x*%SrTe samples show that the electronic absorption edge shifts towards higher energy with *x* increasing from 0 to 6 and remains unchanged afterwards (Fig. 1b). Figure 1c shows that the optical bandgap increases from 0.23 eV in pristine PbTe to 0.34 eV for samples with *x* higher than 5. The larger bandgaps are consistent with successful alloying of the much wider bandgap semiconductor SrTe (*E*_g_=3.5 eV, ref. 22) in PbTe.

The presence of band convergence in the PbTe–SrTe systems was probed with Hall effect measurements (Fig. 1d). Typically, for semiconductors with only a single band contributing to the charge transport, the Hall coefficient (*R*_H_) is nearly temperature independent. The case is very different for p-type lead or tin chalcogenides featuring two or more valence bands^14^,^17^,^23^,^24^,^25^,^26^. In these compounds, as the temperature rises, the L band moves to lower energy while the Σ band remains almost unchanged, resulting in a smaller Δ*E*_L−Σ_ and carrier redistribution between the two sub-bands^14^,^17^,^26^. This change in band structure is reflected in the strong temperature dependence of *R*_H_ which peaks at a temperature when the contribution from the two bands to hole transport is comparable^23^,^26^,^27^. The temperature (*T*_H_) corresponding to the peak of *R*_H_ is a measure of Δ*E*_L−Σ_ and is greatly affected when the band structure is altered for example by alloying^17^,^23^,^26^. For pure p-type PbTe with different hole concentrations, *R*_H_ always peaks at ∼425 K (refs 14, 17; Fig. 1d). However, when PbTe is alloyed with MgTe or MnTe, Δ*E*_L−Σ_ is diminished which is reflected in the lowered *T*_H_ (refs 14, 15, 16). In our current study, we see a similar decreasing trend of *T*_H_ as the SrTe content is increased (Supplementary Fig. 4), indicating that the addition of SrTe decreases Δ*E*_L−Σ_, which is consistent with our theoretical calculations presented below. On the contrary, in our previous report on PbTe–SrTe using equilibrium synthesis, *R*_H_ peak temperature shows negligible changes with increasing SrTe fraction because of the very low solubility of SrTe in PbTe (ref. 7). Therefore, the high-temperature Hall data serves as another diagnostic to confirm the high solubility of SrTe with PbTe achieved in this work.

We also explored the band structure modification of PbTe by alloying with SrTe using first-principles density functional theory (DFT) electronic structure calculations. Since Na serves only as a dopant in PbTe adjusting the carrier concentration, as has been demonstrated by numerical experiment studies and theoretical calculations^28^,^29^,^30^, for the simplicity of calculation, we showed the band structure calculation results of PbTe–SrTe without Na. The DFT band structures of PbTe–SrTe with and without spin–orbit interaction (SOI) are shown in Fig. 1e and Supplementary Fig. 5, respectively, both of which exhibit qualitatively similar trends for *E*_g_ and Δ*E*_L−Σ_ (Supplementary Fig. 6). When the Pb atoms are partially replaced by Sr, the conduction band minimum at the L point of PbTe remains roughly constant in energy while both the L and Σ valence bands move away from the conduction band, leading to an enlarged *E*_g_. As the L band decreases in energy much faster than the Σ band, Δ*E*_L−Σ_ decreases, Fig. 1f. The larger *E*_g_ and smaller Δ*E*_L−Σ_ favourably impact the thermoelectric power factors of the PbTe–*x*%SrTe system, as we present below.

**Figure 2:**
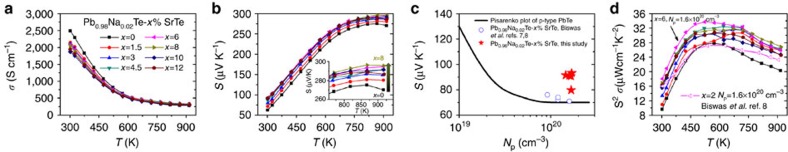
Enhanced electrical properties of Pb_0.98_Na_0.02_Te–*x*%SrTe by band structure modification. (**a**) Electrical conductivity and (**b**) Seebeck coefficient and (**d**) power factor as a function of temperature for Pb_0.98_Na_0.02_Te–*x*%SrTe. Inset of **b** is an expanded view of the Seebeck coefficient of Pb_0.98_Na_0.02_Te–*x*%SrTe between 700–923 K, showing a gradual enhancement of Seebeck coefficient with *x* increasing from 0 to 8.The open symbols in **d** denote Pb_0.98_Na_0.02_Te–2%SrTe under equilibrium processing^8^. (**c**) Room temperature Seebeck coefficient as a function of hole concentration (*N*_p_) for Pb_0.98_Na_0.02_Te–*x*%SrTe. The solid line is the theoretical Pisarenko plot for pure PbTe^14^,^34^. The data of equilibrium prepared samples of Pb_0.98_Na_0.02_Te–*x*%SrTe by Biswas *et al*. are also included for comparison^7^,^8^. The Seebeck coefficients in the non-equilibrium samples are enhanced by ∼30%.

For all samples, the electrical conductivity (Fig. 2a) decreases while the Seebeck coefficient (Fig. 2b) increases with increasing temperature, typical of degenerate conduction. Because of the similar hole concentrations (Supplementary Table 1), the high-temperature electrical conductivity for all samples is similar as the SrTe content is increased from 0 to 12%; in contrast, the Seebeck coefficient shows a considerable enhancement with increasing SrTe. Specifically, the Seebeck coefficient is 62 μV K^−1^ for *x*=0 and goes up to greater than 90 μV K^−1^ for *x*>6 at 300 K (Supplementary Table 1). Also, the Seebeck coefficient of the Pb_0.98_Na_0.02_Te control sample shows a clear downturn ∼800 K attributed to bipolar diffusion^31^. In contrast, the SrTe-containing samples, show continuously increasing Seebeck coefficient up to 923 K suggesting negligible bipolar conduction. This behaviour is consistent with the enlarged bandgap (Fig. 1c) which inhibits the thermal activation of minority carriers at elevated temperatures.

The room temperature Hall carrier concentrations (*N*_p_) and hole mobilities (μ_H_) for all samples are given in Supplementary Table 1. We estimated the carrier effective mass (m*) of each sample from the measured *S* and *N*_p_ considering a single parabolic band^32^ for simplicity. The m* increases gradually from 0.82 *m*_e_ to 1.42 *m*_e_ (*m*_e_ is the free electron mass) with increasing SrTe content from 0 to 6 mol%. We have proposed above that the alloying of SrTe causes convergence between the two valence bands (L and Σ) of PbTe. The degeneracy (*N*_v_) of the L band of PbTe is 4 while that of the Σ band is much larger, *N*_v_=12 (refs 9, 17). When the L and Σ bands are sufficiently close in energy (for example, by SrTe alloying), the effective *N*_v_ becomes 12–16 rather than 4 for the L band or 12 for the Σ band^9^,^14^. As m* is related to *N*_v_ through the relation m*=(*N*_v_)^2/3^*m*_b_* (*m*_b_* is the band effective mass)^9^,^33^, this valence band convergence effect (increase of *N*_v_) significantly increases m* by employing more degenerate valleys in the hole transport without seriously deteriorating *μ*_H_ (Supplementary Table 1).

The valence band convergence caused by the much higher SrTe alloying achieved with the non-equilibrium processing is reflected in the enhancement of the Seebeck coefficient as shown by the Pisarenko relation between *S* and *N*_p_ (Fig. 2c). The solid line is the established theoretical Pisarenko plot^14^,^34^ for PbTe. We observe that the Seebeck coefficients for our heavily Sr-alloyed PbTe samples are much higher than predicted by the solid line for PbTe and behave very similar to Mg- or Mn-doped PbTe (refs 14, 15, 16; Supplementary Fig. 7). On the contrary, the Seebeck coefficients of the lightly alloyed PbTe–SrTe samples of Biswas *et al*.^7^,^8^ prepared using equilibrium synthesis fall exactly on the Pisarenko plot for PbTe.

These results further demonstrate that much higher amounts of SrTe successfully alloyed into PbTe contribute to the marked enhancement of the Seebeck coefficient over a broad temperature range (Fig. 2b). Therefore, the power factors (*S*^2^*σ*) of the Pb_0.98_Na_0.02_Te–*x*%SrTe samples in this study are remarkably enhanced, with the maximum values exceeding 30 μW cm^−1^ K^−2^ around 500 K (Fig. 2d). These values are the highest reported for any p-type PbTe (refs 7, 8, 14, 15, 16; Supplementary Fig. 8), including the equilibrium synthesized Pb_0.98_Na_0.02_Te–*x*%SrTe by Biswas *et al*.^8^ with similar hole densities (<25 μW cm^−1^ K^−2^). We also compared the temperature-dependent power factors of Na-doped PbTe alloyed with 3 mol% MgTe, MnTe and SrTe in this study (within the solid solution region where alloying-induced valence band convergence is taking effect; Supplementary Fig. 9). Although having a similar *N*_p_, the 3 mol% SrTe sample displays significantly higher power factor than the other two (note that their Seebeck coefficients at room temperature are quite similar, specifically, 80 μV K^−1^ for 3%SrTe, 88 μV K^−1^ for 3% MnTe (ref. 16) and 89 μV K^−1^ for 3% MgTe (ref. 15)). The reason could be that the smaller radius contrast between Pb^2+^ (1.19 Å) and Sr^2+^ (1.32 Å) (for comparison, the radii of Mg^2+^ and Mn^2+^ are 0.86 and 0.83 Å, respectively) makes the scattering of carriers from crystal defects less significant. As an evidence of this conjecture, we list the room temperature carrier mobilities for the three samples as follows: 69, 65 and 78 cm^2^ V^−1^ s^−1^ for 3% MgTe (ref. 15), MnTe (ref. 16) and SrTe alloyed PbTe, respectively. This additional advantage from crystal chemistry, along with the valence band convergence in the band structure by SrTe alloying, endows the PbTe–SrTe system with superior electrical properties.

### Thermal transport and valence band alignment

**Figure 3:**
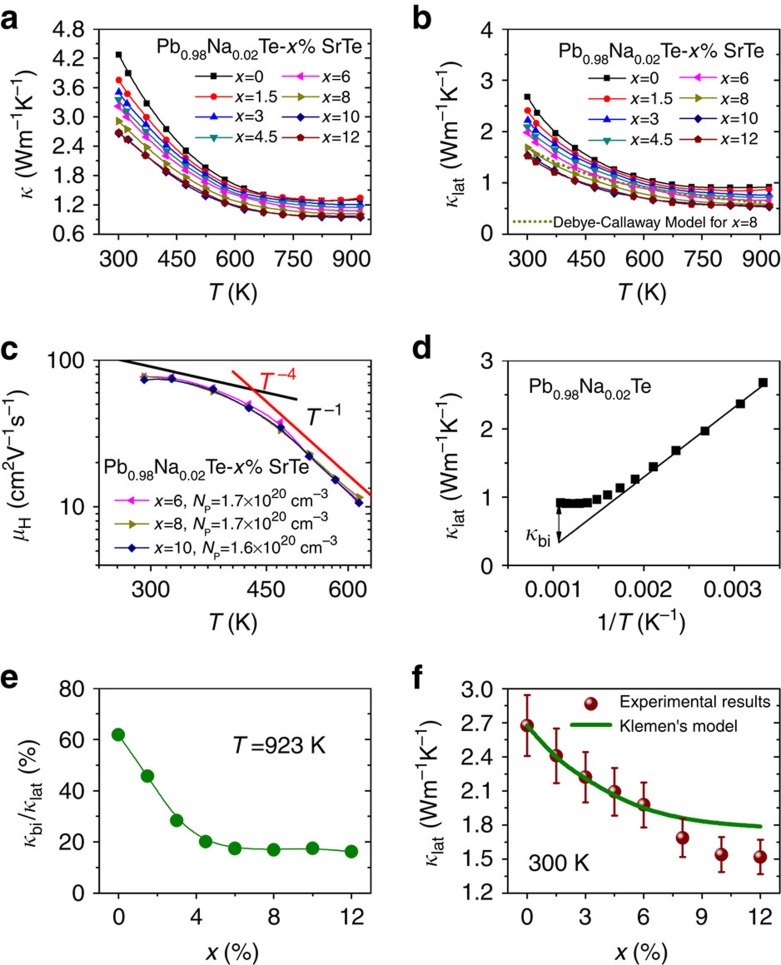
Decoupling of electron and phonon transport and suppression of bipolar conduction in Pb_0.98_Na_0.02_Te–*x*%SrTe. (**a**) Total and (**b**) lattice thermal conductivity as a function of temperature, showing a steady decrease with increasing SrTe fraction up to 10 mol%. The dotted line represents the simulated lattice thermal conductivities of *x*=8 sample using Debye-Callaway model^36^. (**c**) Temperature-dependent Hall mobilities of nanostructured Pb_0.98_Na_0.02_Te–*x*%SrTe (*x*=6, 8 and 10) with similar hole densities. The black and red lines represent the power-law temperature dependence of *T*^−1^ and *T*^−4^, respectively. Negligible changes in mobilities are observed as SrTe second phase amount is increased. (**d**) An example describing how to extract bipolar thermal conductivity (*κ*_bi_) of Pb_0.98_Na_0.02_Te by linearly fitting the lattice thermal conductivity versus reciprocal temperature. The solid line indicates that *κ*_lat_ is inversely proportional to temperature. (**e**) The ratio of *κ*_bi_ to *κ*_lat_ as a function of SrTe content at 923 K, indicating largely suppressed bipolar thermal conductivity with increasing SrTe content due to enlarged bandgap. (**f**) Room temperature lattice thermal conductivities of Pb_0.98_Na_0.02_Te–*x*%SrTe as a function of SrTe fraction. 10% Error bars are applied. The green line is a modelled lattice thermal conductivity using a modified Klemens model^37^,^38^. Departure from the Klemens model at high SrTe fraction is ascribed to the nanostructures.

Both the total (*κ*, Fig. 3a) and lattice thermal conductivity (*κ*_lat_, Fig. 3b) of PbTe are considerably suppressed upon the addition of SrTe. The 10% SrTe sample exhibits the lowest room temperature *κ*_lat_ of 1.5 W m^−1^ K^−1^, which drops to 0.5 W m^−1^ K^−1^ at 923 K. However, the SrTe nanostructuring has little impact on the carrier mobilities in Pb_0.98_Na_0.02_Te–*x*%SrTe (*x*=0–12). Figure 3c shows hole mobilities (*μ*_H_) as a function of temperature for *x*=6, 8 and 10 samples (all are nanostructured) with similar hole concentrations of 1.6–1.7 × 10^20^ cm^−3^. Clearly the changes of *μ*_H_ with increasing SrTe amounts are negligible. This is attributed to the favourable energy alignment of the valence bands of PbTe and SrTe as reported earlier^7^. Moreover, for all samples, the mobilities follow a power-law temperature dependence of *T*^−1^ from 300 to 400 K and *T*^−4^ from 400 to 700 K. The fast degradation of mobilities at elevated temperature in p-type PbTe-based compounds arises from the contribution of the heavy holes in the Σ valence band.

For semiconductors with Umklapp scattering as the dominant phonon scattering mechanism, *κ*_lat_ should vary as 1/*T* before any bipolar diffusion becomes significant^25^,^26^,^32^,^35^, which is the case in PbTe (ref. 14). *κ*_lat_ starts to deviate from such a relationship when the contribution from bipolar diffusion (*κ*_bi_) is significant (Fig. 3d). Figure 3e shows the ratio of *κ*_bi_ to *κ*_lat_ at 923 K as a function of SrTe content for Pb_0.98_Na_0.02_Te–*x*%SrTe. The high content of SrTe strongly inhibits the bipolar diffusion because of the enlarged bandgap.

Using the Debye-Callaway model^36^ we were able to simulate the lattice thermal conductivities of the PbTe–8%SrTe sample (see Supplementary Methods for simulation details and Supplementary Table 2 where the related parameters for simulation are listed) and figure out the contributions from different scattering mechanisms to the lattice thermal conductivity by examining the relaxation time versus the normalized frequency plot (Supplementary Fig. 10). We find that the simulated lattice thermal conductivities agree fairly with the experimental ones (Fig. 3b). In addition, the contribution from strain, induced by point defects, to the lattice thermal conductivity is more significant at lower frequency while nanoprecipitates and point defects are more important phonon scattering sources at medium phonon frequencies. We also modelled the room temperature lattice thermal conductivities of Pb_0.98_Na_0.02_Te–*x*%SrTe using a modified Klemens model^37^,^38^. This model takes into account both the mass and strain field contrasts and has been demonstrated extremely useful for disordered alloys^25^,^26^,^39^,^40^. In this model, Pb_0.98_Na_0.02_Te is treated as a perfect crystal. The input parameters for the calculation are: Debye temperature of 136 K (ref. 41), average sound velocity of 1,770 m s^−1^ (ref. 41), and an adjustable parameter (related to elastic properties) of 65 (ref. 42). The modelled lattice thermal conductivities shown in Fig. 3f agree fairly well with the experimental results when *x*<6 (solid solution region), suggesting that alloy scattering plays a large role in the reduction of lattice thermal conductivity. An apparent departure of the experimental data from the Klemens model is observed for samples with higher SrTe content (*x*>6). This deviation is attributed to the strong scattering of phonons from the ubiquitous SrTe nanostructures^7^,^8^,^11^,^43^,^44^,^45^.

**Figure 4:**
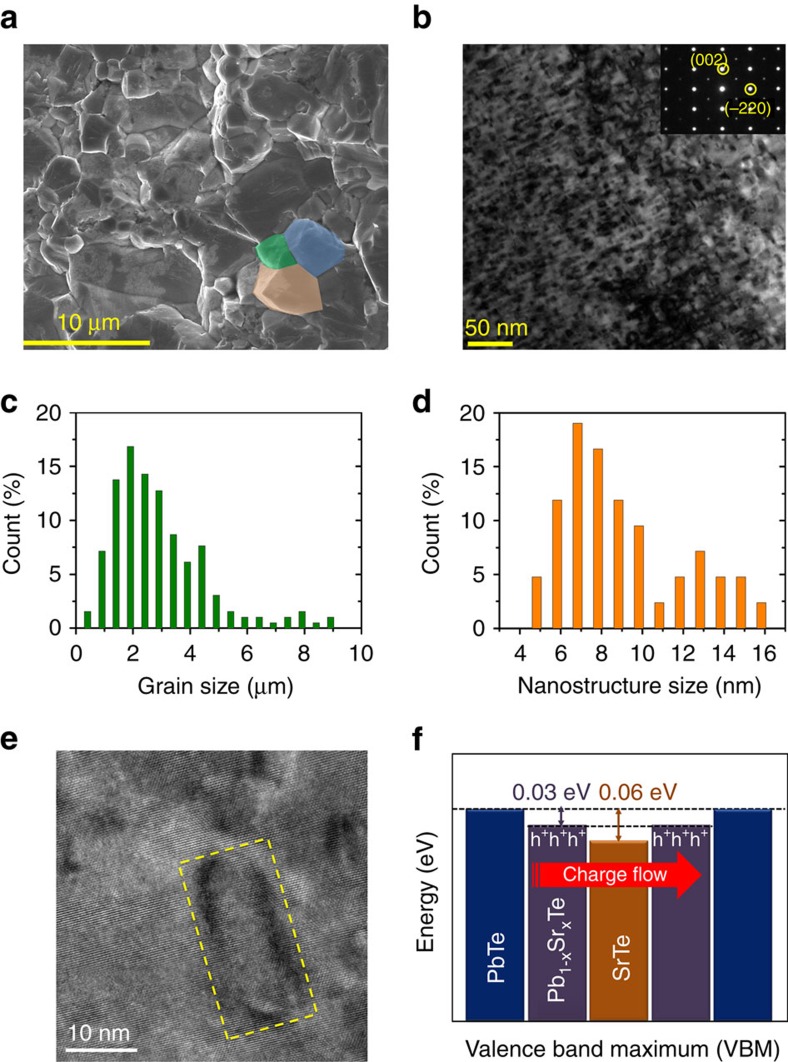
All-scale hierarchical architectures and matrix/precipitate valence band alignment in Pb_0.98_Na_0.02_Te–8%SrTe. (**a**) SEM image showing micron sized grains in the specimen. The length of the scale bar is 10 μm. The coloured areas (orange, olive and blue) are drawn as a guide to the eyes to show the grain sizes of the order of several microns. (**b**) Low magnification TEM image of specimen along the [110] zone axis, as indicated by the selected area electron diffraction pattern in the inset image. Large density nanoscale precipitates with darker contrast are observed. The length of the scale bar is 50 nm. (**c**) Size distribution of the mesoscale grains based on SEM images like **a**. (**d**) Size distribution of the nanoscale precipitates based on HRTEM images. (**e**) HRTEM image of the precipitates highlighted by the dashed yellow window showing coherent interfaces between precipitate and matrix. The length of the scale bar is 10 nm. (**f**) Schematic representation of the alignment of the valence band (VB) energies of SrTe precipitates in the PbTe and Pb_1−*x*_Sr_*x*_Te matrix, based on our theoretical first-principles calculations.

Microstructural and compositional information was obtained using scanning electron microscopy (SEM), (scanning) transmission electron microscopy ((S)TEM) equipped with energy dispersive spectroscopy and secondary ion mass spectrometry (SIMS). Figure 4a shows a SEM image on a freshly fractured surface of Pb_0.98_Na_0.02_Te–8%SrTe, with grain sizes ranging from 1 to 3 μm on average, as indicated in Fig. 4c. The low magnification TEM image presents high-density nanoprecipitates with darker contrast along the [110] zone axis, as shown in Fig. 4b. The inset shows a selected area electron diffraction pattern that exhibits single crystal diffraction pattern indicating the endotaxial relationship of the precipitates with the PbTe matrix. Moreover, Na, Sr, Pb and Te are all spatially uniformly distributed under SIMS (Supplementary Fig. 11). In Fig. 4d, the size distribution of the nanoscale precipitates shows a peak of 7 nm along the [110] zone axis. The HRTEM image in Fig. 4e reveals coherent interfaces between precipitates and matrix, highlighted by dashed yellow rectangular box. These interfaces enable effective phonon scattering and minimize hole scattering, and account for the low thermal conductivity, high mobility and high-power factors in our samples.

Our valence band alignment calculations indicate that the valence band energy offset (Δ*E*_V_) between PbTe and SrTe is only 0.06 eV with the valence band maximum (VBM) of PbTe lying slightly higher than that of SrTe (Fig. 4f). When Sr substitutes for Pb in PbTe, in addition to the band convergence discussed above, the VBM of PbTe is further lowered because of the formation of solid solution Pb_1−*x*_Sr_*x*_Te (Fig. 1e,f; Supplementary Fig. 5). A previous study on PbTe–MgTe by Pei *et al*.^15^ suggested that a shift of the peak *R*_H_ to lower temperature by 70 K (which is also the case in our study, Supplementary Fig. 4) represents an energy reduction of 0.03 eV between L and Σ bands. Applying this argument, we estimate that Δ*E*_V_ is only 0.03 eV (nearly aligned) between SrTe precipitates and Pb_1−*x*_Sr_*x*_Te matrix, Fig. 4f. This matrix/precipitate band alignment preserves the high hole mobility in nanostructured systems^7^,^8^,^46^,^47^.

**Figure 5:**
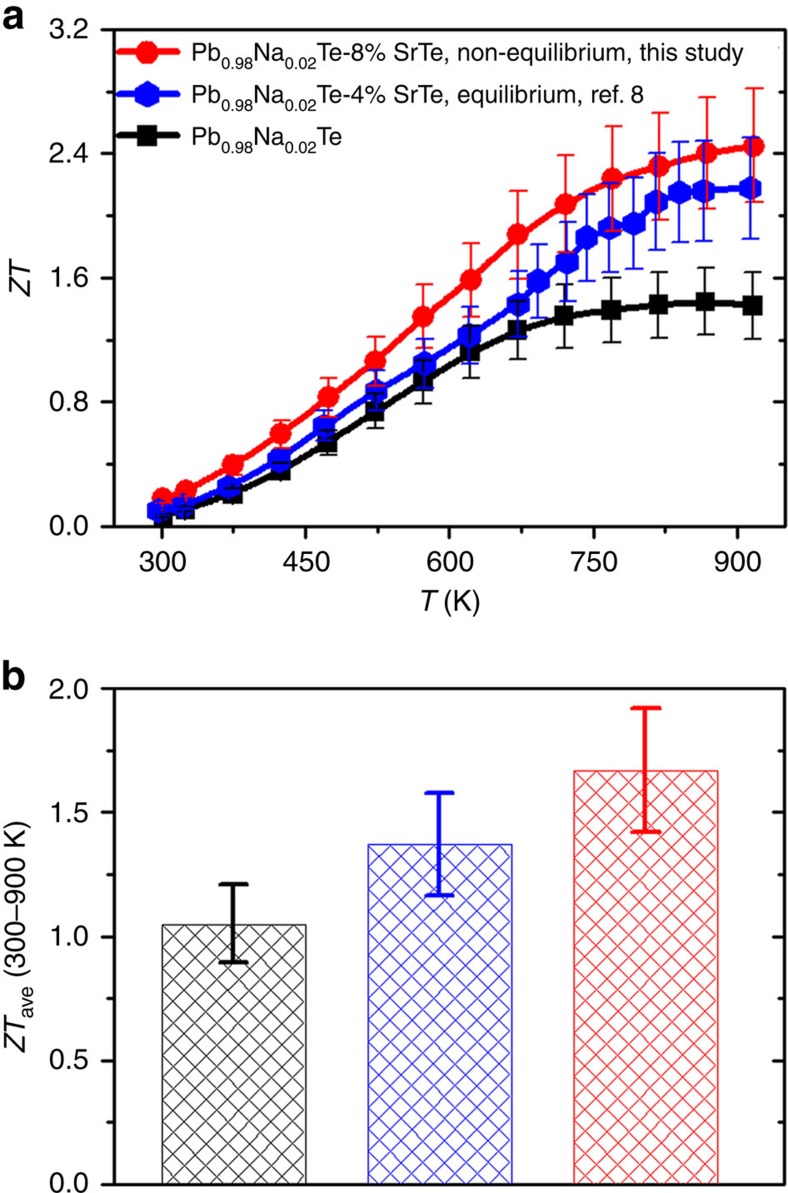
Thermoelectric figure of merit *ZT* of Pb_0.98_Na_0.02_Te–*x*%SrTe. (**a**) Comparison of *ZT* values of Pb_0.98_Na_0.02_Te–*x*%SrTe (*x*=0 and 8) in this study, with those of Biswas *et al*.^8^ (**b**) Comparison of *ZT*_ave_ in the range of 300–900 K for the above three samples. The black, blue and red bars represent the control sample Pb_0.98_Na_0.02_Te, the Pb_0.98_Na_0.02_Te–4%SrTe sample by equilibrium synthesis and the Pb_0.98_Na_0.02_Te–8%SrTe sample by non-equilibrium synthesis, respectively. Note that 15% error bars are indicated.

With increasing SrTe content dissolved in PbTe, we have demonstrated the highest power factors in PbTe-based systems while preserving the all-scale architectures that give low thermal conductivities. The combination of charge and thermal transport properties presented above leads to high thermoelectric performance for Pb_0.98_Na_0.02_Te–*x*%SrTe (see their *ZT* values in Supplementary Fig. 12), with a record high *ZT* of 2.5 at 923 K for *x*=8. This is nearly 80% improvement over the control sample *x*=0 (*ZT*=1.4) and 15% higher than the sample by Biswas *et al*. (*ZT*=2.2) (Fig. 5).

We also note that the magnitude of *ZT* enhancement in this study with respect to the previous work is especially noticeable at *T*<800 K while at elevated temperature (800–923 K) this enhancement becomes less significant. In PbTe the change of the energy levels of the L and Σ valence bands with increasing temperature is an unusual phenomenon which causes band convergence. According to recent studies^48^,^49^, in PbTe itself, the temperature-driven L-Σ valence band convergence occurs at 700–780 K. Simply applying this argument and considering that further SrTe alloying decreases the energy separation between the two bands (Fig. 1e; Supplementary Fig. 4), one would expect a slightly lower than 700–780 K band convergence temperature as schematically shown in Supplementary Fig. 13. Therefore, at very high temperature (*T*>800 K) the thermoelectric performance of PbTe–SrTe system would not benefit from the temperature-driven band convergence more than PbTe itself does. However, the enlarged bandgap (*E*_g_) of PbTe by alloying with SrTe could play a significant role in improving the thermoelectric performance at elevated temperature (*T*>800 K), due to the suppression of bipolar diffusion. For example, the Seebeck coefficient of PbTe–SrTe shows no sign of downturn up to 900 K (Fig. 2b) while its bipolar thermal conductivity is largely decreased (Fig. 3c). When the temperature is not high enough to result in temperature-driven band convergence (for example, *T*<600 K), the SrTe alloying-induced band convergence (decrease of Δ*E*_L−Σ_) makes the contribution from the heavy Σ valence band to the Seebeck coefficient become noteworthy. As a result, one can observe a large enhancement of Seebeck coefficient of PbTe–SrTe as compared to pure PbTe, see Pisarenko relationship between Seebeck coefficients and carrier concentrations (Fig. 2c). Correspondingly, the maximum power factors achieved in our PbTe–SrTe samples exceed 30 μW cm^−1^ K^−2^ at around 600 K (Fig. 2d). They are the highest reported for p-type PbTe. In addition, the *ZT*s at low temperature are significantly enhanced (Fig. 5).

The average *ZT* (*ZT*_ave_), which is important for device applications, in the range of 300–900 K, is 1.67 for the high performing sample in the present study. In comparison, *ZT*_ave_ is 1.05 for PbTe and 1.37 for the lightly alloyed samples of Biswas *et al*.^8^ The high performing Pb_0.98_Na_0.02_Te–8%SrTe sample showed negligible changes of thermoelectric properties during the multiple heating–cooling measurement cycles and after a 15 days of vacuum annealing at 823 K, suggesting an excellent thermal stability (Supplementary Figs 14 and 15). More solid evidence of this stability comes from the unchanged lattice parameters and bandgaps before and after the vacuum annealing (Supplementary Fig. 16). We believe that the non-equilibrium solid is in a meta-stable state, but the stability is not compromised at the highest temperatures that we probed in this study.

**Figure 6:**
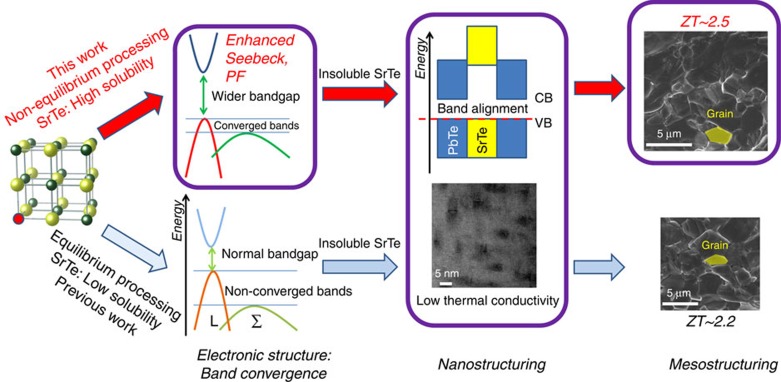
Roadmap towards record high thermoelectric performance in Pb_0.98_Na_0.02_Te–*x*%SrTe polycrystals by non-equilibrium synthesis. Lower route (previous work is Biswas *et al*.^8^): equilibrium synthesis of Pb_0.98_Na_0.02_Te–*x*%SrTe has a low solubility limit of SrTe in PbTe (less than 1 mol%). No valence band convergence is present. The high *ZT* of 2.2 is ascribed to the all-scale hierarchical architectures (atomic scale point defects by Na doping, nanoscale SrTe precipitates and mesoscale grains by SPS treatment) for extremely low thermal conductivity and valence band alignment for unaffected power factors. Upper route (this work): non-equilibrium synthesis of Pb_0.98_Na_0.02_Te–*x*%SrTe dissolves a high fraction of SrTe (5 mol%) in PbTe. This causes strong valence band convergence and bandgap enlargement, leading to higher power factors. Both routes share the all-scale hierarchical architectures that produce very low thermal conductivities. All together these effects lead to a record high *ZT* of 2.5. The lengths of the scale bars are 5 nm and 5 μm in the Nanostructuring and Mesostructuring panels, respectively.

Figure 6 illustrates the difference between equilibrium and non-equilibrium processing in the PbTe–SrTe system. The lower half of Fig. 6 shows how equilibrium processing gives low SrTe solubility (<1%) and leads to band alignment, nanostructuring but not band convergence. This route gives a *ZT* of 2.2 at 913 K. The upper part of Fig. 6 shows how non-equilibrium processing can give much higher dissolved fraction of SrTe (5 mol%). In addition to the band alignment and nanostructuring, the higher alloying fraction of SrTe converges the two valence bands (L and Σ) and enlarges the bandgap, leading to much higher power factors and improved performance. This tight integration of all these properties into a single material leads to record high *ZT* values for Pb_0.98_Na_0.02_Te–8%SrTe of 2.5 at 923 K. We further demonstrate that a peak *ZT* of 2.5 can be achieved at a relatively lower temperature around 800 K by optimizing the Na concentration (Supplementary Fig. 17), making PbTe–SrTe system as a robust candidate for thermoelectric power generation.

## Methods

### Synthesis

High purity Pb wire (99.99%, American Elements, US), Sr chunk (99.9%, Sigma-Aldrich, USA), Na chunk (99.999%, Sigma-Aldrich, US) and Te shot (99.999%, 5N Plus, Canada) were used as the starting materials to synthesize 15 g of Pb_1−*y*_Na_*y*_Te–*x*%SrTe (*y*=0.02, *x*=0, 1.5, 3, 4.5, 6, 8, 10, 12; *x*=8, *y*=1%, 1.25%, 1.5%, 1.75; in mole ratio). Desired amounts of Pb, Sr, Na and Te were weighed and loaded into 13 mm diameter carbon coating silica tubes under an N_2_-filled glove box. The tubes were then evacuated to a residual pressure of ∼10^−4^ torr, flame-sealed, slowly heated up to 1,423 K in 11 h, soaked at this temperature for 12 h and subsequently ice water quenched to room temperature. The tubes containing the molten samples were periodically shaken to ensure the homogeneity of the compositions. The quenched ingots were further vacuum annealed at 873 K for 3 days. To probe into the bandgap variation of PbTe as a function of SrTe content using infrared spectroscopy (IR), a series of PbTe–*x*%SrTe ingots without Na doping (∼5 g in mass for each, *x*=0, 2, 4, 6 and 8, in mole ratio) were also synthesized using the same route. All samples synthesized in this study are all single phase compounds within the detection limit of powder X-ray detection limit (Supplementary Figs 1, 18 and 19). For a typical experiment the following amounts were used: Pb (8.7440, g, 42.2010, mmol), Na (0.0198, g, 0.8612, mmol), Sr (0.3018, g, 3.4450, mmol) and Te (5.9343, g, 46.5072, mmol) were used to prepare 15 g of Pb_0.98_Na_0.02_Te–8%SrTe.

### Densification

The annealed cast ingots of Pb_0.98_Na_0.02_Te–*x*%SrTe were ground into fine powders using agate mortar under an N_2_-filled glove box, put inside a 12.7 mm diameter graphite die and densified by SPS (SPS-211LX, Fuji Electronic Industrial Co., Ltd.) at 823 K for 5 min under an axial compressive stress of 60 MPa in vacuum. Highly dense disk-shaped pellets with dimensions of 12.7 mm diameter and ∼10 mm thickness were obtained (Supplementary Table 3).

### Electron microscopy and X-ray diffraction

(Scanning) transmission electron microscopy (S/TEM) and STEM energy dispersive spectroscopy experiments investigations were carried out using a JEOL 2100F microscope operated at 200 kV. The thin TEM specimens were prepared by conventional methods, including cutting, grinding, dimpling, tripod, with minimal duration of Ar-ion milling with a liquid N_2_ cooling stage. Samples pulverized with an agate mortar were used for powder X-ray diffraction. SIMS was performed with PHI TRIFT III ToF-SIMS. The sample was prepared by mechanical polishing on SiC paper with ethanol and then on cloth with oil-based SiC slurry. A 15 kV Gallium ion beam was used in the secondary ion mass spectrometry. After initial spectrum collection, Ga ion was sputtered on a 100 × 100 μm surface area of the sample for 60 s to clean out the surface contamination. Spectrum and mapping images were collected using a raster size (size of the sputter area on the sample) of 10 × 10 μm. The powder diffraction patterns were obtained with Cu K_α_ (*λ*=1.5418 Å) radiation in a reflection geometry on an Inel diffractometer operating at 40 kV and 20 mA and equipped with a position-sensitive detector. The lattice parameter was obtained using a Rietveld refinement method (Fig. 1a).

### Electrical properties

The obtained SPS-processed pellets were cut into bars with dimensions 12 × 3 × 3 mm^3^ for simultaneous measurement of the Seebeck coefficient and electrical conductivity using an Ulvac Riko ZEM-3 instrument under a low-pressure helium atmosphere from room temperature to 923 K. Samples were spray coated with boron nitride spray to minimize outgassing except where needed for electrical contact with the thermocouples, heater and voltage probes. The uncertainty of the Seebeck coefficient and electrical conductivity measurements is 5%, which is widely accepted for Ulvac instruments^50^.

### Thermal conductivity

Highly dense SPS-processed pellets were cut and polished into a square shape of 6 × 6 × 2 mm^3^ for thermal diffusivity measurements. The samples were coated with a thin layer of graphite to minimize errors from the emissivity of the material. The total thermal conductivity was calculated from *κ*_tot_=*D*·*C*_p_·*d*, where the thermal diffusivity coefficient (*D*) was measured using the laser flash diffusivity method in a Netzsch LFA457, and the density (*d*) was determined using the dimensions and mass of the sample. Heat capacity (*C*_p_) is estimated from the relation *C*_p_/*k*_B_ per atom atom=3.07+(4.7 × 10^−4^ × (*T*−300))^15^,^16^, which is obtained by fitting the experimental data reported by Blachnik^51^ within an uncertainty of 2% above room temperature. The thermal diffusivity data were analysed using a Cowan model with pulse correction. The measured densities of all the samples range between 7.9 and 8.0 g cm^−3^ or are above 97% of the theoretical densities (Supplementary Table 3). The uncertainty of the thermal conductivity is estimated to be within 8% (ref. 50), considering all the uncertainties from *D*, *C*_p_ and *d*. The lattice thermal conductivity (*κ*_lat_) was calculated by subtracting the electrical thermal conductivity (*κ*_el_) from *κ*_tot_ using a Wiedemann-Franz relationship *κ*_el_=*L*·*σ*·*T*, where *L* is Lorenz number which can be obtained by fitting the Seebeck coefficient to the reduced chemical potential^32^,^52^. The values of *C*_p_, *D* and *L* values for all the samples in this study can be found in Supplementary Figs 20 and 21. The combined uncertainty for all measurements involved in the calculation of *ZT* is <15%. Unless otherwise noted, all the thermoelectric properties were measured perpendicular to the sintering pressure direction, although no directional anisotropy effects (<3%) were observed in the charge transport properties.

### Hall measurements

The high-temperature Hall measurement was performed on a homemade apparatus (University of Michigan) in an argon atmosphere. The Hall resistance was monitored with a Linear Research AC Resistance Bridge (LR-700), with constant magnetic fields of ±1 T applied by using an Oxford Superconducting magnet. The effective carrier concentration (*N*_p_) was estimated using the relationship *N*_p_=1/*eR*_H_, where *e* is the elemental charge, and *R*_H_ is the Hall coefficient. The Hall mobility (*μ*_H_) was calculated using the relationship *μ*_H_=*σR*_H_ with *σ* being the electrical conductivity obtained from the ZEM-3 instrument. It should be mentioned here that the use of two different instruments to deduce carrier mobility data may cause some uncertainties.

### Infrared spectroscopy

Room temperature optical diffuse reflectance measurements were performed on finely ground powders to probe optical energy gap of the series. The spectra were collected in the mid-IR range (6,000–400 cm^−1^) using a Nicolet 6700 FT-IR spectrometer. The reflectance versus wavelength data generated, were used to estimate the bandgap by converting reflectance to absorption data according to Kubelka–Munk equations: α/S=(1−*R*)^2^/(2*R*), where *R* is the reflectance, *α* and *S* are the absorption and scattering coefficients, respectively.

### Band structure calculations

DFT calculations of pristine, stoichiometric PbTe, and Sr-doped PbTe were carried out. The calculations were performed using the generalized gradient approximation with PBE^53^ functional for the exchange−correlation functional and projector augmented wave potentials as implemented in Vienna *ab initio* Simulation Package (VASP)^54^. All the atomic positions are relaxed until the forces exerted on the atoms are less than 0.001 eV A^−1^. The calculated lattice constant of PbTe is 6.43 Å, which is very close to experimental result 6.463 Å. All structures are fully relaxed with respect to cell vectors and cell-internal positions. For the Pb and Sr species, the 5*d* electrons and 4*p* 5*s* states are respectively treated as valence states. The total energies were numerically converged to ∼3 meV per cation using a basis set energy cutoff of 400 eV and dense k-meshes corresponding to 4,000 k-points per reciprocal atom in the Brillouin zone.

To investigate the movements of the conduction band and valence bands (L and Σ bands) with added Sr ions, we considered 3 × 3 × 3 supercells of NaCl-type Pb_27_Te_27_. For the isovalent doping of Sr, we consider a single Sr impurity (∼3.7% and 7.4% additions of Sr, Pb_26_Sr_1_Te_27_ and Pb_25_Sr_2_Te_27_) with Sr substituting for Pb. The symmetry of the original primitive cell was changed by the substitution defects in PbTe, thus, for the purposes of a more direct comparison with PbTe we transformed the eigenstates for defect structures into a so-called effective band structure in the primitive Brillouin zone of the parent compound PbTe using a spectral decomposition method^55^,^56^. Using this approach, we are able to calculate the energy level of the L point and Σ line and the corresponding energy differences for the supercells with defects. Note that the band structures are calculated by considering both with SOI (Fig. 1e) and non-SOI (Supplementary Fig. 5), and similar trends were observed in both cases.

To assess the relative band alignments of the second phase SrTe and MgTe with host phase PbTe we utilize the findings of Van de Walle and Neugebauer, who demonstrated a universal alignment of the electronic transition level of hydrogen in a wide range of materials including semiconductors, insulators and even aqueous solutions^57^. Hence, to infer the band alignment, we compute the energies of H defects in the rock salt compounds of interest, assume alignment between these H energies, and then extract the band alignment of the compounds. To align the valance band maximum position of each system, we consider the defect formation energies of various charge states of interstitial *H*^*q*^ (*q*=−1, 0, 1) by placing *H* in the host material with a 128 atom supercell, calculating the total energy of this structure, and subtracting the energy of the corresponding pure host material, hydrogen chemical potential, and electron chemical potential:


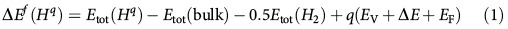


where *E*_V_ and *E*_F_ are VBM and Fermi level (relative to the VBM). To select the most favourable interstitial *H* binding sites in host materials, multiple binding configurations are calculated. The electrostatic potential correction term Δ*E* is calculated by inspecting the potential in the supercell far from the impurity and aligning it with the electrostatic potential in bulk^58^.

### Data availability

The authors declare that the data supporting the findings of this study are available within the article and its Supplementary Information files, or from the corresponding authors upon request.

## Additional information

**How to cite this article:** Tan, G. *et al*. Non-equilibrium processing leads to record high thermoelectric figure of merit in PbTe–SrTe. *Nat. Commun.* 7:12167 doi: 10.1038/ncomms12167 (2016).

## Supplementary Material

Supplementary Information Supplementary Figures 1-21, Supplementary Tables 1-3, Supplementary Methods and Supplementary References.
