# Supplementary material for: Non-equilibrium processing leads to record high thermoelectric figure of merit in PbTe–SrTe
Source: Nat Commun. 2016 Jul 26;7:12167. doi: 10.1038/ncomms12167 (PMC4963473; doi:10.1038/ncomms12167)
Supplement: Supplementary Information — Supplementary Figures 1-21, Supplementary Tables 1-3, Supplementary Methods and Supplementary References. [file ncomms12167-s1.pdf]

Supplementary Figures

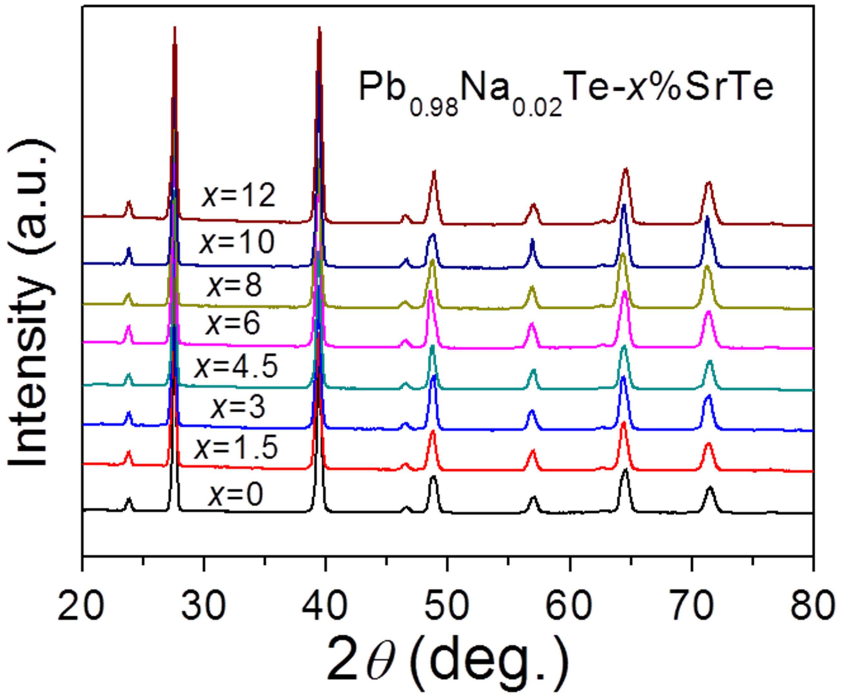

Supplementary Figure 1 | Phase structure study of the synthetic samples. Powder X-ray diffraction patterns of $Pb_{0.98}Na_{0.02}Te$-*x*%SrTe samples.

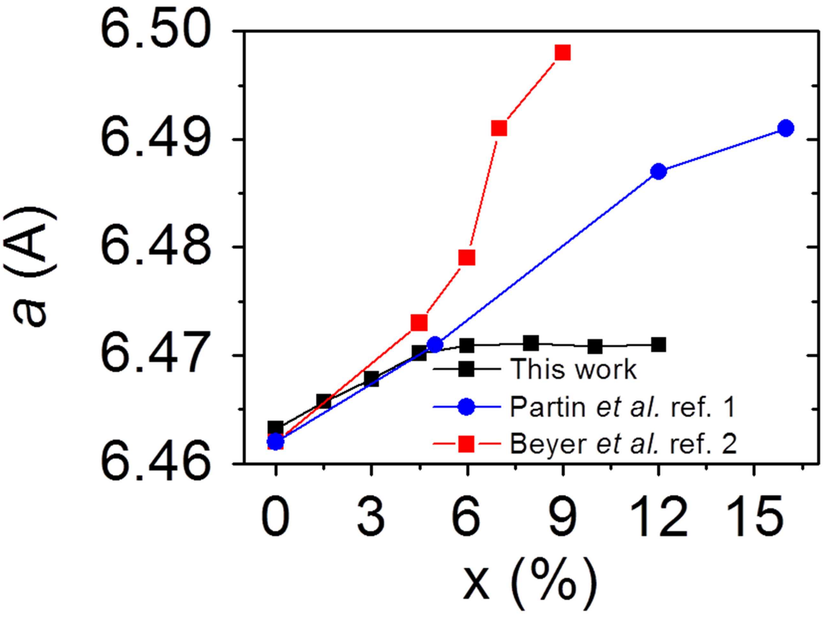

Supplementary Figure 2 | Composition dependence of lattice parameter. Comparison of variations of lattice parameters of PbTe-*x*%SrTe bulk materials in this study and thin films[1,2] in previous works. A very similar composition dependence of lattice parameter among different samples suggests the alloying behavior between PbTe and SrTe.

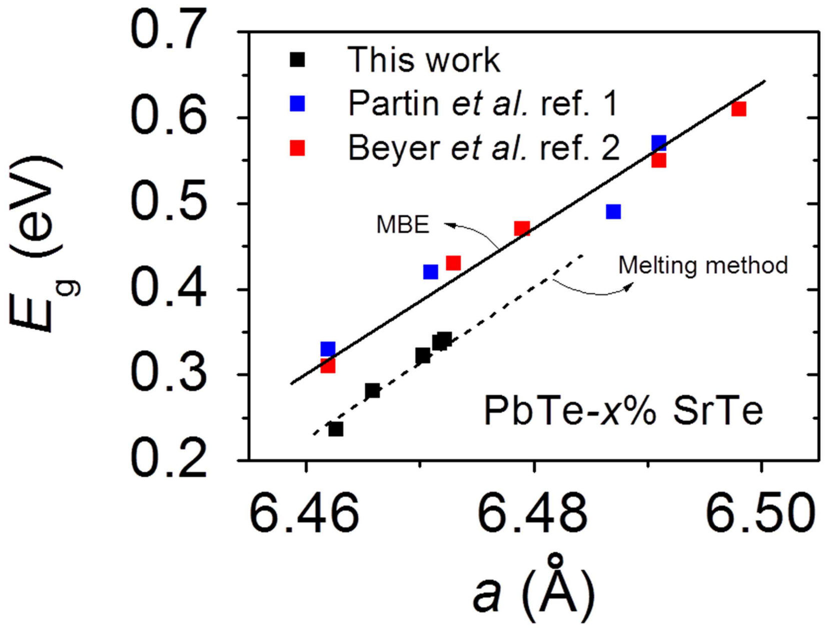

Supplementary Figure 3 | Band gap as a function of lattice parameter. Comparison of band gaps (E_g) as a function of lattice parameters (a) between PbTe-x%SrTe bulk materials in this study prepared by melting method and thin films[1,2] in previous works grown by molecular beam epitaxy (MBE). Similar slopes of E_g *versus* a plots between bulks and films suggest that non-equilibrium ice water quenching synthesis is as effective as MBE in producing high alloying fraction of SrTe in PbTe.

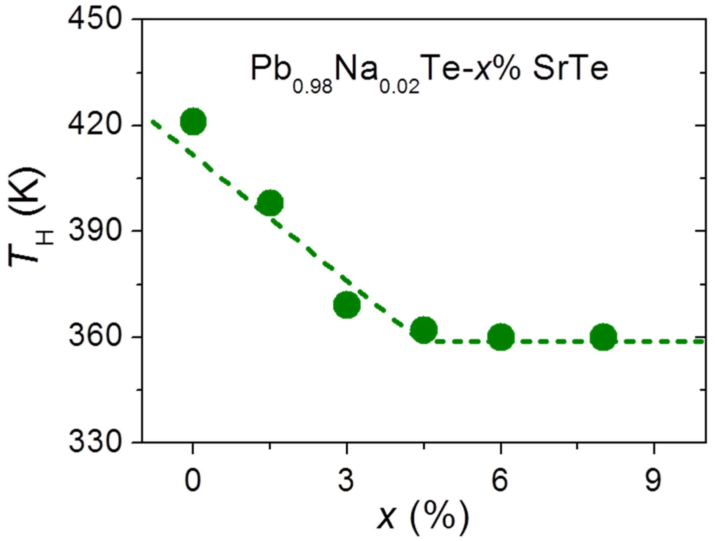

Supplementary Figure 4 | Alloying PbTe with SrTe lowers the Hall peak temperature. The Hall coefficient peaking temperature (T_H) as a function of SrTe fraction for $Pb_{0.98}Na_{0.02}Te$-x%SrTe. The dotted line is fitted on the basis of Vegard's law. The cross-point coordinate of (4.51, 358) indicates that the maximum alloying fraction of SrTe in PbTe is around 5 mol.%. The T_H is shifted to lower temperature by ~65 K (from ~423 K for x=0 to 358 K for x = 6 and beyond).

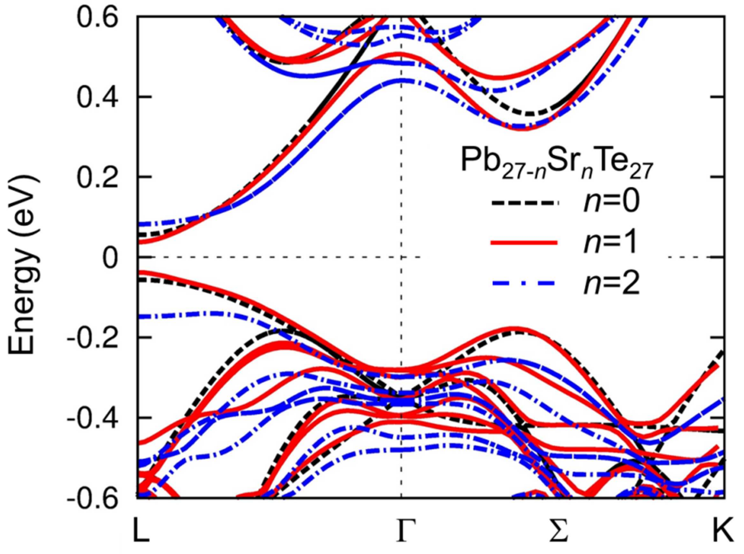

Supplementary Figure 5 | Electronic structure of PbTe-SrTe with spin-orbit interaction. First principles band structure calculations of $Pb_{27-n}Sr_nTe_{27}$ (n=0, 1 and 2), where spin-orbit interaction (SOI) is considered.

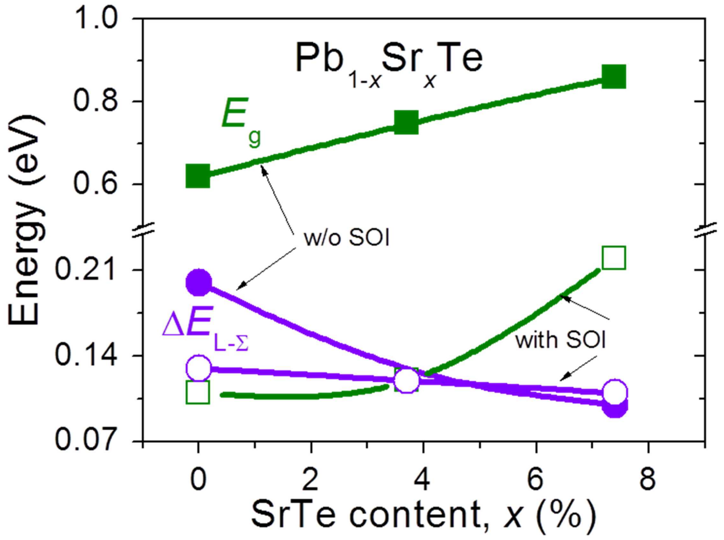

Supplementary Figure 6 | Variation of band gap and energy difference between the two valence bands of PbTe as SrTe alloying fraction is increased. Theoretically calculated band gaps (E_g) and energy offsets ($\Delta E_{L-\Sigma}$) between the two valence bands of PbTe as a function of SrTe doping concentration. Solid symbols are results without spin-orbit interaction (w/o SOI) while open symbols are for those with SOI (Supplementary Fig. 5). In the case of w/o SOI, E_g increases while $\Delta E_{L-\Sigma}$ decreases rapidly and significantly with increasing SrTe content, highly consistent with the experiment trends we observed. However, when SOI is considered, we note that the increase of Eg and decrease of $\Delta E_{L-\Sigma}$ become much less significant with increasing SrTe content. As SOI is important in band structure calculation of heavy element alloys like PbTe, we believe that there is a big challenge to theory when dealing with complex real materials. It deserves further study in the future.

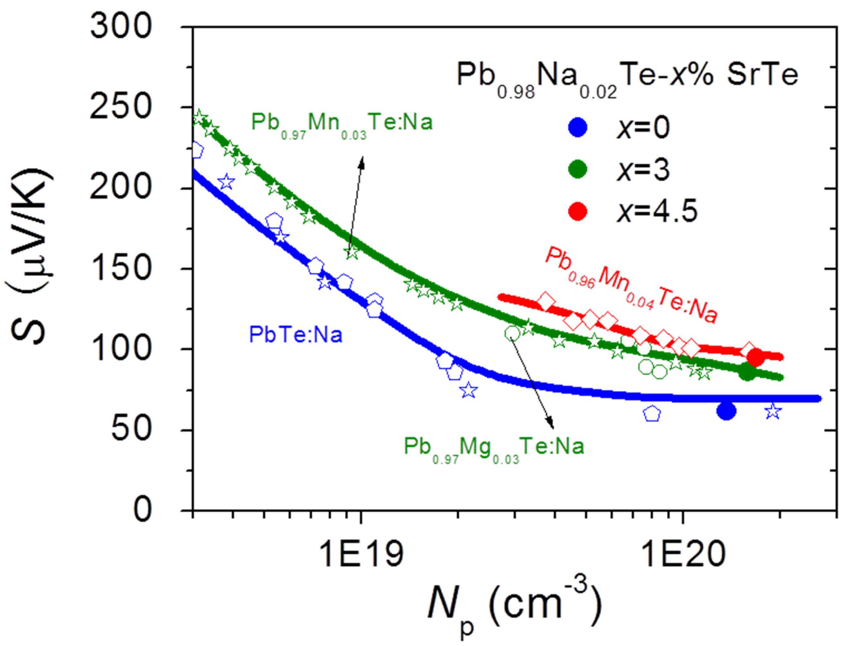

Supplementary Figure 7 | Pisarenko plot of p-type PbTe based compounds. The blue line is the theoretical Pisarenko plot[3,4] for PbTe while the green and red lines are experimental ones for 3% and 4% Mn or Mg-doped PbTe, respectively[3,5,6]. The S-N_p relation of PbTe with varying Na concentrations[7,8] can be well described by the blue line, which is also the case in our 2 mol.% Na-doped PbTe, demonstrating the pure dopant nature of Na. The significantly higher Seebeck coefficients of Mn- or Mg-doped PbTe in comparison to those of PbTe at similar carrier concentrations are a consequence of valence band convergence that has been discussed in detail previously[3,5,6]. We observe that the Seebeck coefficients for our 3 and 4.5 mol.% Sr-doped PbTe quenched samples are also much higher than predicted by the blue line for PbTe and fall exactly on the green and red lines for 3 and 4 mol.% Mn or Mg-doped PbTe, respectively.

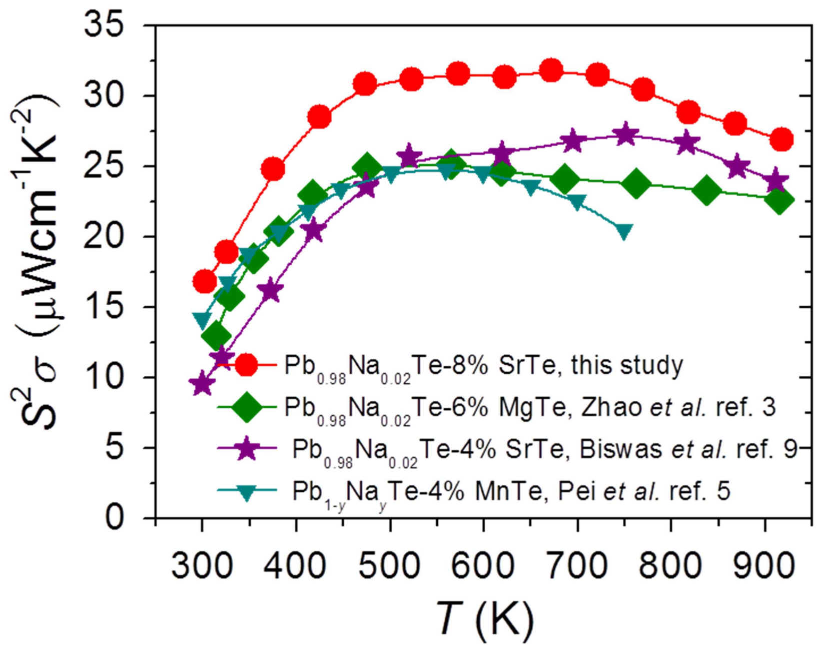

Supplementary Figure 8 | Non-equilibrium synthesized PbTe-SrTe display the highest power factors. Comparison of power factors for several p-type PbTe-based materials: $Pb_{0.98}Na_{0.02}Te$-8%SrTe (this study), $Pb_{0.98}Na_{0.02}Te$-6%MgTe (Zhao *et al.*, ref. 3), $Pb_{0.98}Na_{0.02}Te$-4%SrTe (Biswas *et al.*, ref. 9) and $Pb_{1-y}Na_yTe$-4%MnTe (Pei *et al.*, ref. 5), among which the first one clearly outperforms all others in the entire working temperature range.

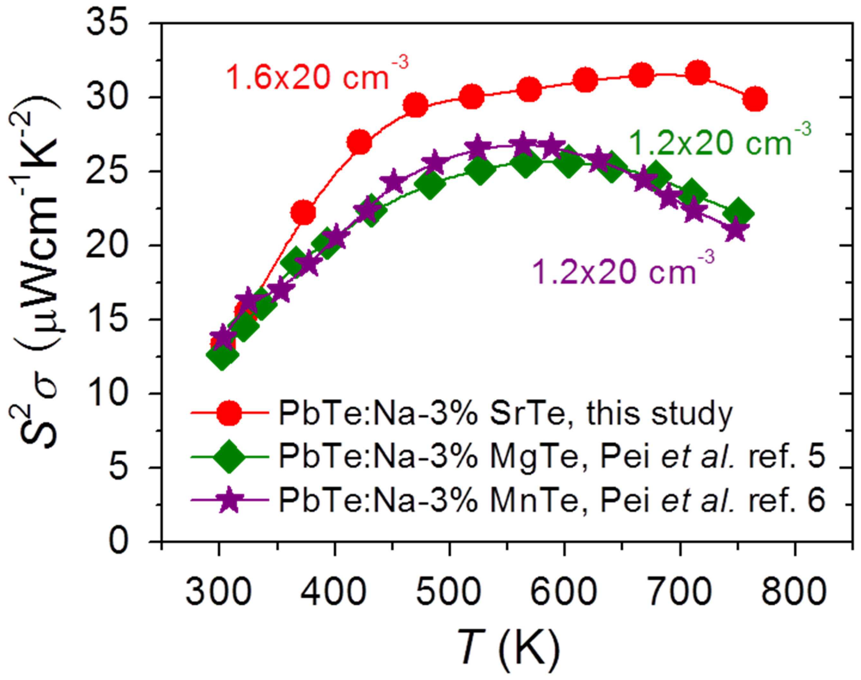

Supplementary Figure 9 | High power factor benefiting from least crystal structure distortion. Comparison of power factors for p-type PbTe alloyed with 3% SrTe (in this study), 3% MgTe (Pei *et al.*, ref. 5) and 3% MnTe (Pei *et al.*, ref. 6) with comparable hole concentrations.

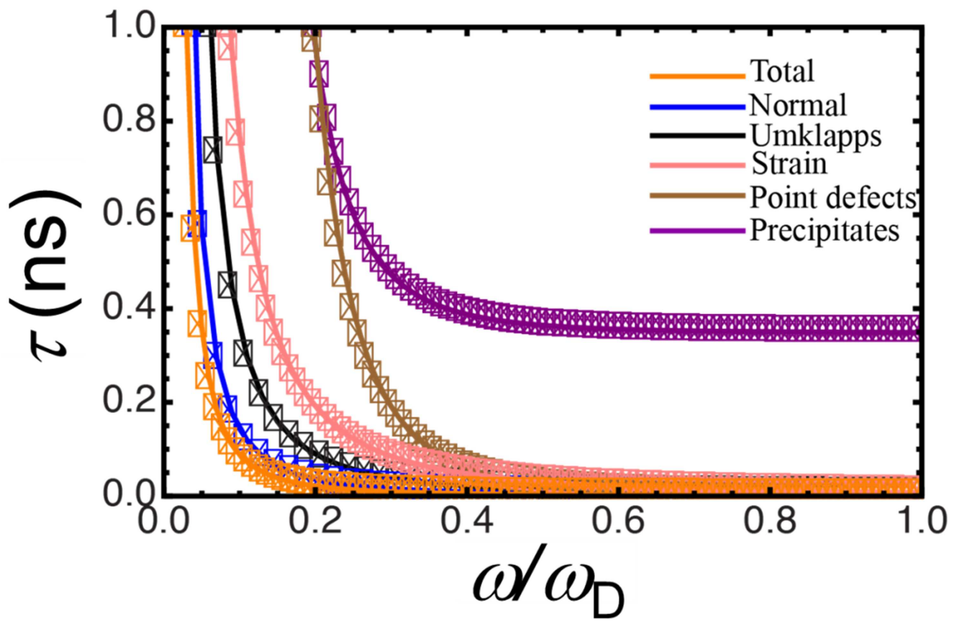

Supplementary Figure 10 | Calculation of relaxation time for PbTe-8%SrTe. Relaxation time *versus* the normalized frequency for $Pb_{0.98}Na_{0.02}Te$-8%SrTe at 300 K. The phonon frequency (ω) is normalized to the Debye frequency (ω_D).

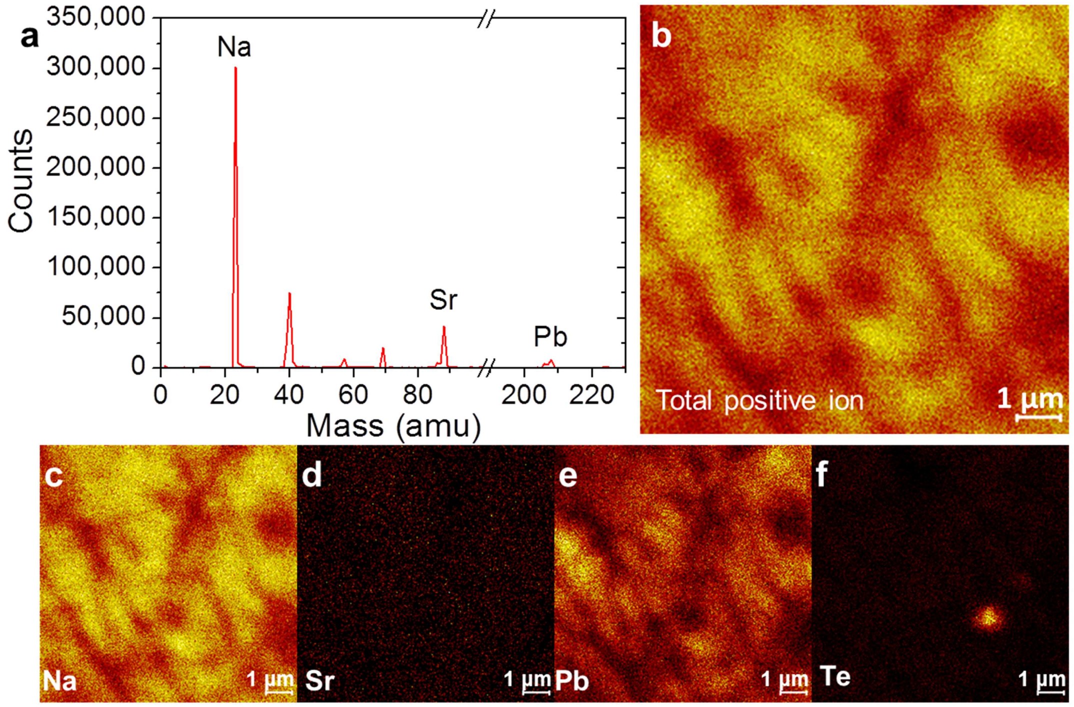

Supplementary Figure 11 | Secondary ion mass spectrometry performed on $Pb_{0.98}Na_{0.02}Te$-8%SrTe. (**a**) The positive ion spectrum: Na, Sr and Pb were found in this spectrum. (**b**)-(**f**) shows the element mapping for: total positive ion, Na ion, Sr ion, Pb ion and Te ion, respectively. Na, Sr, Pb and Te are all spatially uniformly distributed. The contrast in the maps is due to the surface roughness of the sample.

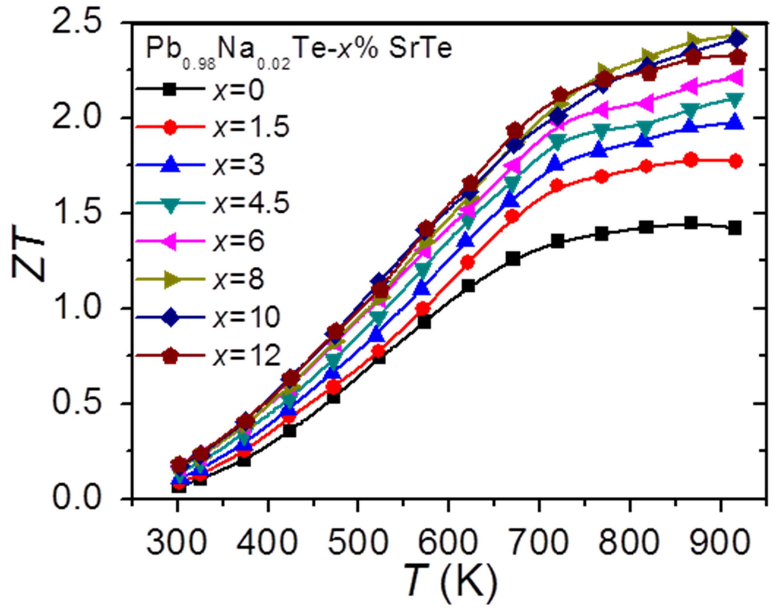

Supplementary Figure 12 | *ZT* values of $Pb_{0.98}Na_{0.02}Te$-*x*%SrTe. Thermoelectric figure of merit *ZT* as a function of temperature for $Pb_{0.98}Na_{0.02}Te$-*x*%SrTe.

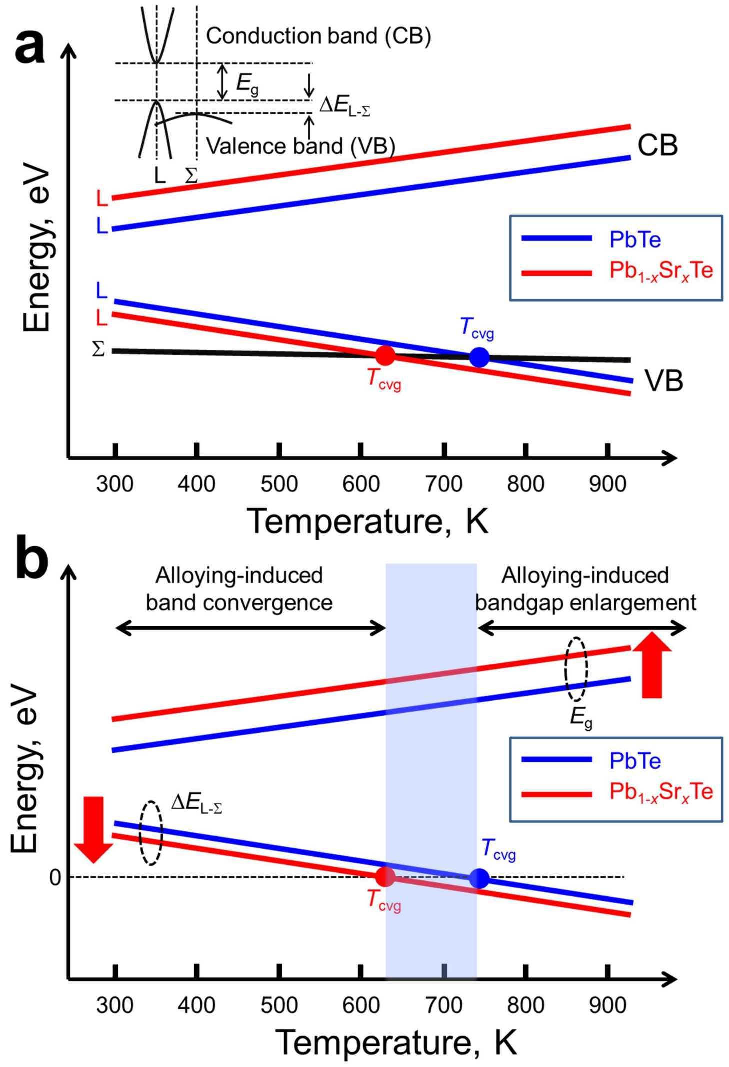

Supplementary Figure 13 | Schematic diagram showing the temperature effects on the electronic structures of PbTe and PbTe-SrTe. Inset of (**a**): room temperature band structure of PbTe, a direct band gap (E_g) forming at L point; L and Σ valence bands (VB) being separated by an energy difference of $\Delta E_{L-\Sigma}$. The blue and red lines represent the temperature dependent electronic structure energy levels of PbTe and PbTe-SrTe, respectively. With increasing temperature, the conduction band (CB) moves towards higher energy while both the L and Σ VBs move towards lower energy, leading to enlarged E_g. Since L VB moves faster than Σ VB, $\Delta E_{L-\Sigma}$ decreases with increasing temperature, namely temperature-driven band convergence. A convergence of L and Σ VBs occurs at T_{cvg}=700-780 K(refs. 10,11). Alloying with SrTe pushes the CB of PbTe to higher energy and L and Σ VBs to lower energy. Since L VB energy decreases more than Σ VB, $\Delta E_{L-\Sigma}$ is lowered, namely alloying-induced band convergence. Correspondingly, T_{cvg} is decreased. (**b**) Changes of the E_g and $\Delta E_{L-\Sigma}$ with temperature for PbTe (blue lines) and PbTe-SrTe (red lines). Alloying induced band convergence works best before temperature-driven band convergence occurs while after L and Σ valence bands are converged, the alloying-induced band gap enlargement serves as the mechanism to maintain high thermoelectric performance.

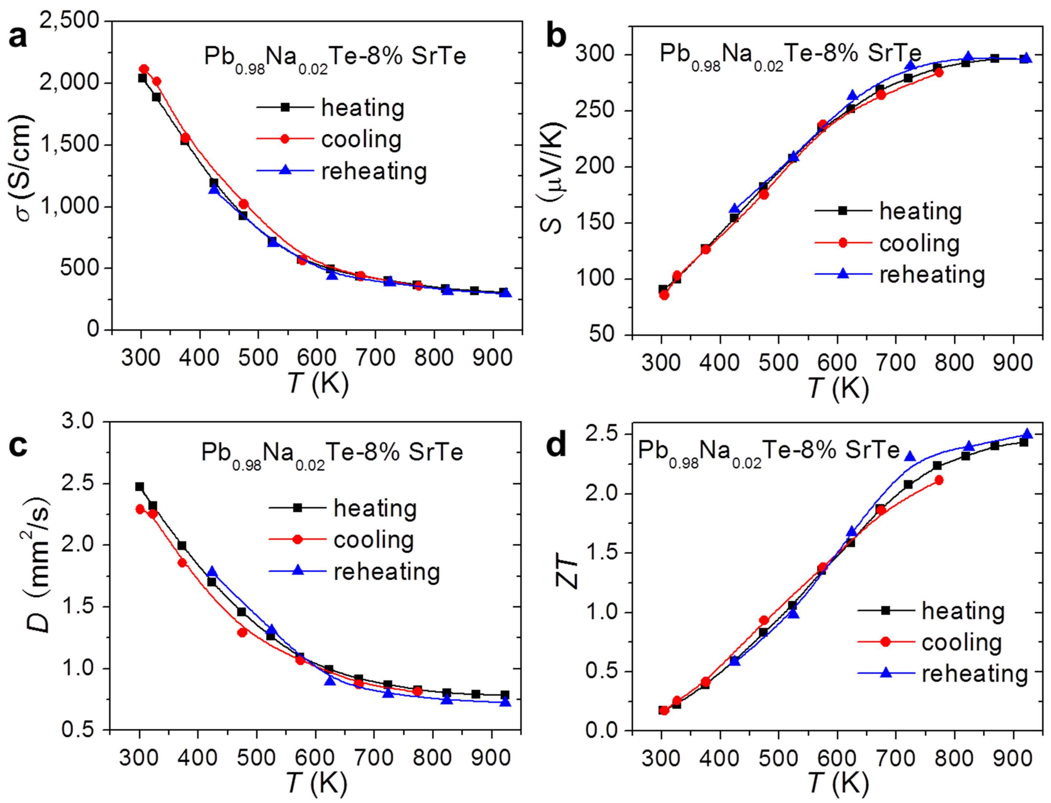

Supplementary Figure 14 | Stability evaluation of the high performance $Pb_{0.98}Na_{0.02}Te$-8%SrTe sample from heating-cooling cycles. (**a**) Electrical conductivity, (**b**) Seebeck coefficient, (**c**) thermal diffusivity, and (**d**) *ZT* value in heating (black squares), cooling (red circles) and reheating (blue triangles).

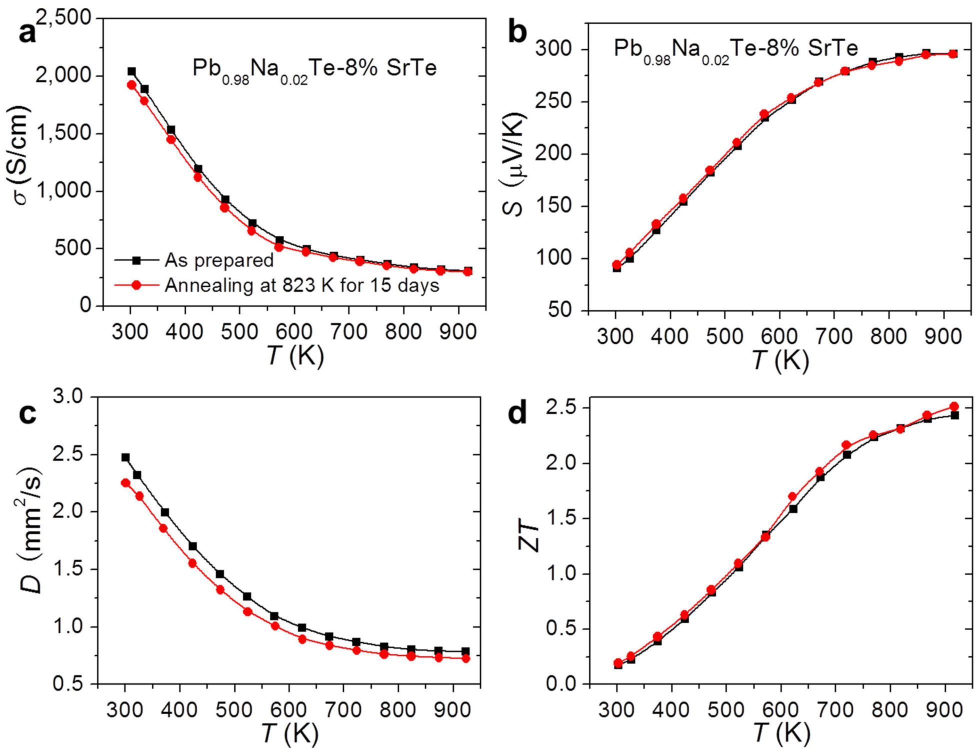

Supplementary Figure 15 | Stability evaluation of the high performance $Pb_{0.98}Na_{0.02}Te$-8%SrTe sample through annealing. (**a**) Electrical conductivity, (**b**) Seebeck coefficient, (**c**) thermal diffusivity, and (**d**) *ZT* value before and after 823 K and 15 d vacuum annealing treatment.

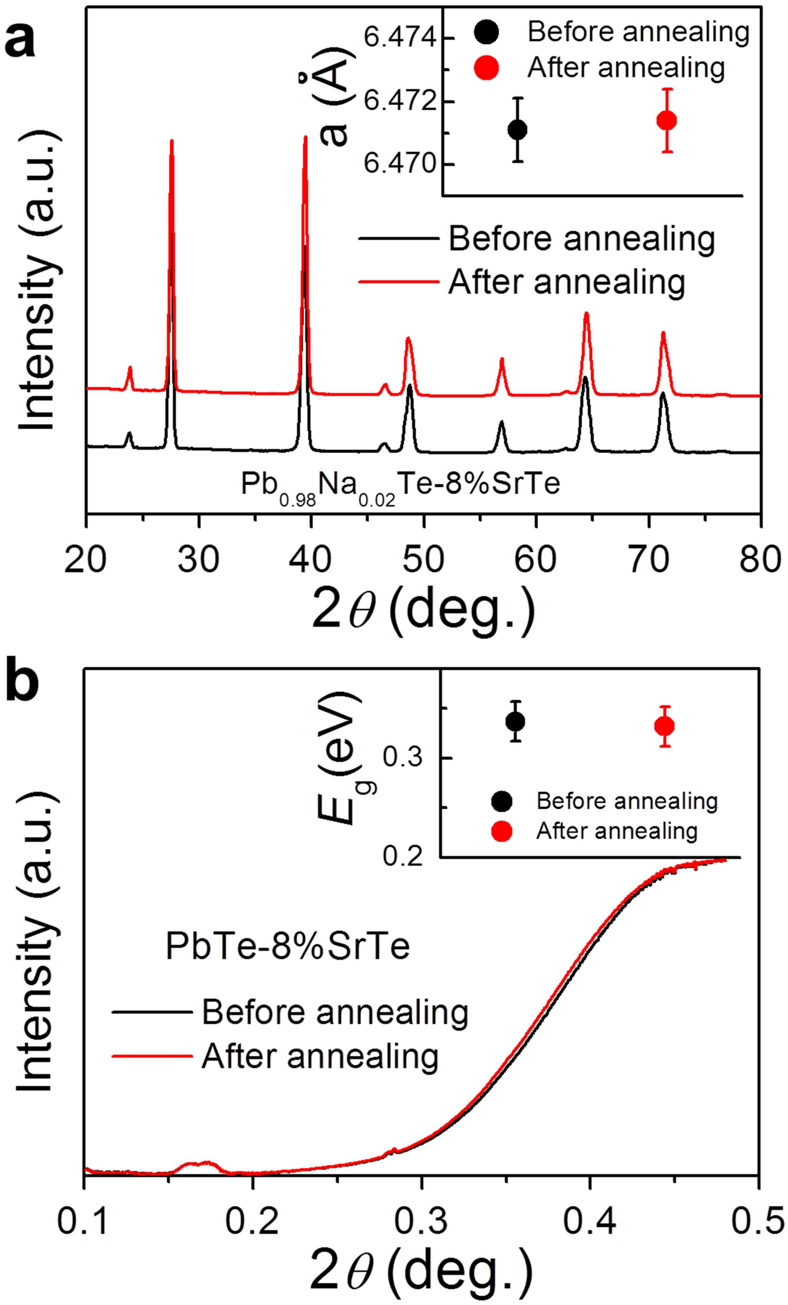

Supplementary Figure 16 | Lattice parameter and band gap variations of PbTe-8%SrTe before and after annealing treatment. (**a**) PXRD of $Pb_{0.98}Na_{0.02}Te$-8%SrTe before and after 823 K-15 d vacuum annealing treatment. Inset: the change of corresponding lattice parameters where 0.001 Å error bar is given. (**b**) Infrared absorption spectra of PbTe-8%SrTe before and after 823 K-15 d vacuum annealing treatment. Inset: the change of corresponding band gaps where 0.01 eV error bar is displayed.

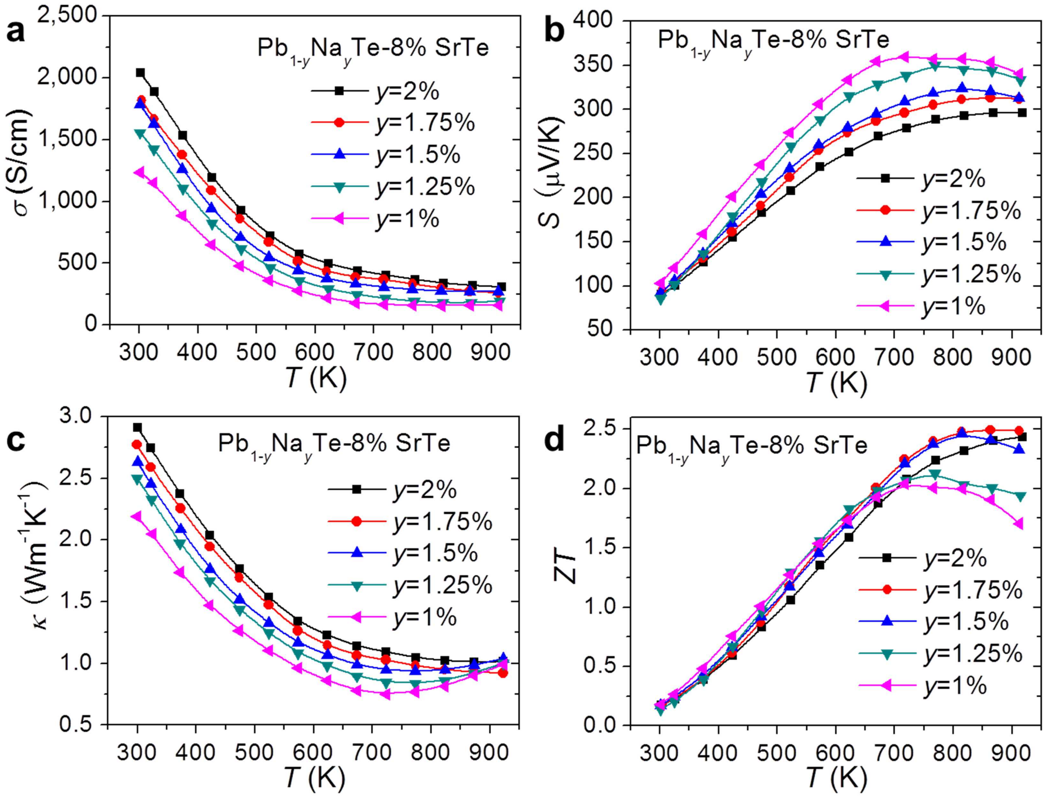

Supplementary Figure 17 | Carrier concentration optimization of $Pb_{1-y}Na_yTe$-8%SrTe (*y*=1-2%). (**a**) Electrical conductivity, (**b**) Seebeck coefficient, (**c**) thermal conductivity, and (**d**) *ZT* values. As expected, with decreasing Na content, the electrical conductivity decreases while the Seebeck coefficient increases because of the decrease of hole concentration. The samples with lower Na dopant concentration do have better performance at lower temperature but due to the stronger bipolar conduction their *ZT*s are degraded at elevated temperature. The 1.5% and 1.75% Na-doped samples have a peak *ZT* of 2.5 at a relatively low temperature around 800 K.

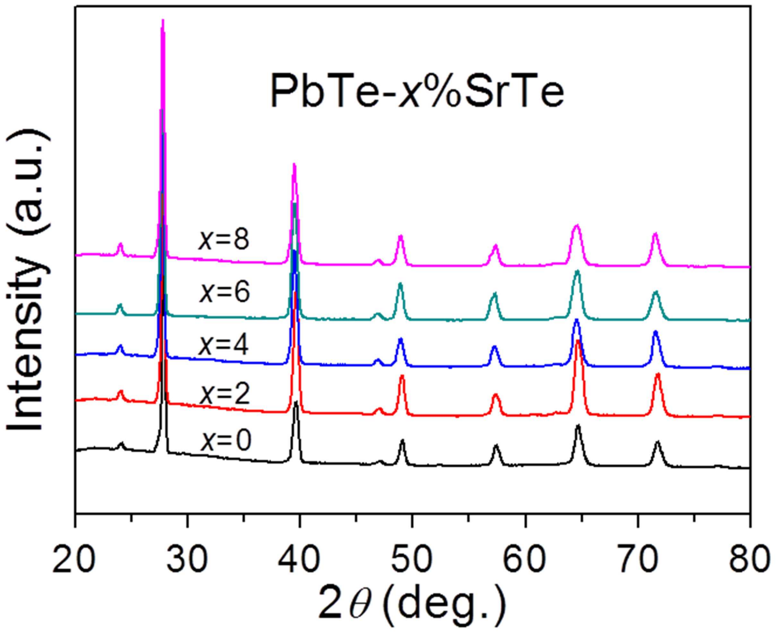

Supplementary Figure 18 | Phase purity examination of PbTe-*x*%SrTe. Powder X-ray diffraction patterns of PbTe-*x*%SrTe samples. They are all single phase compounds crystalizing the rocksalt structure within the detection limit of laboratory X-ray.

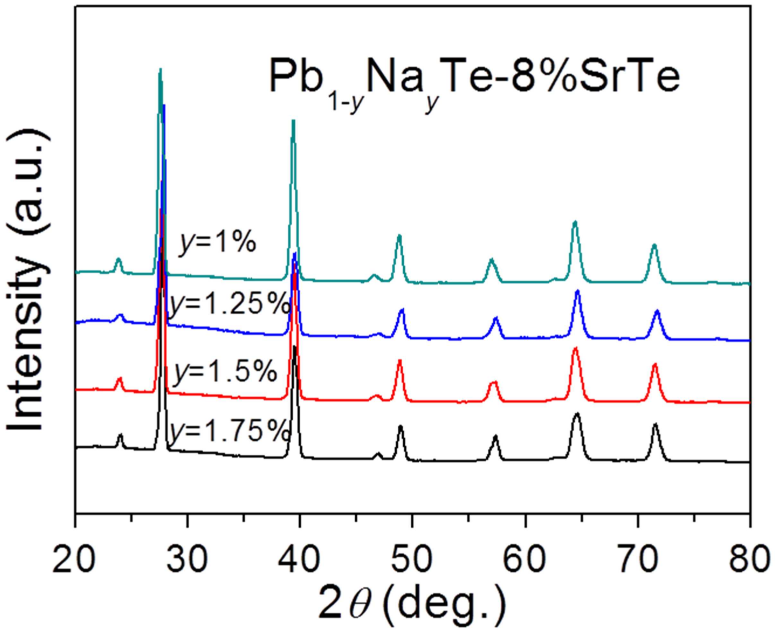

Supplementary Figure 19 | Phase purity examination of $Pb_{1-y}Na_yTe$-8%SrTe. Powder X-ray diffraction patterns of $Pb_{1-y}Na_yTe$-8%SrTe samples. They are all single phase compounds crystalizing the rocksalt structure within the detection limit of laboratory X-ray.

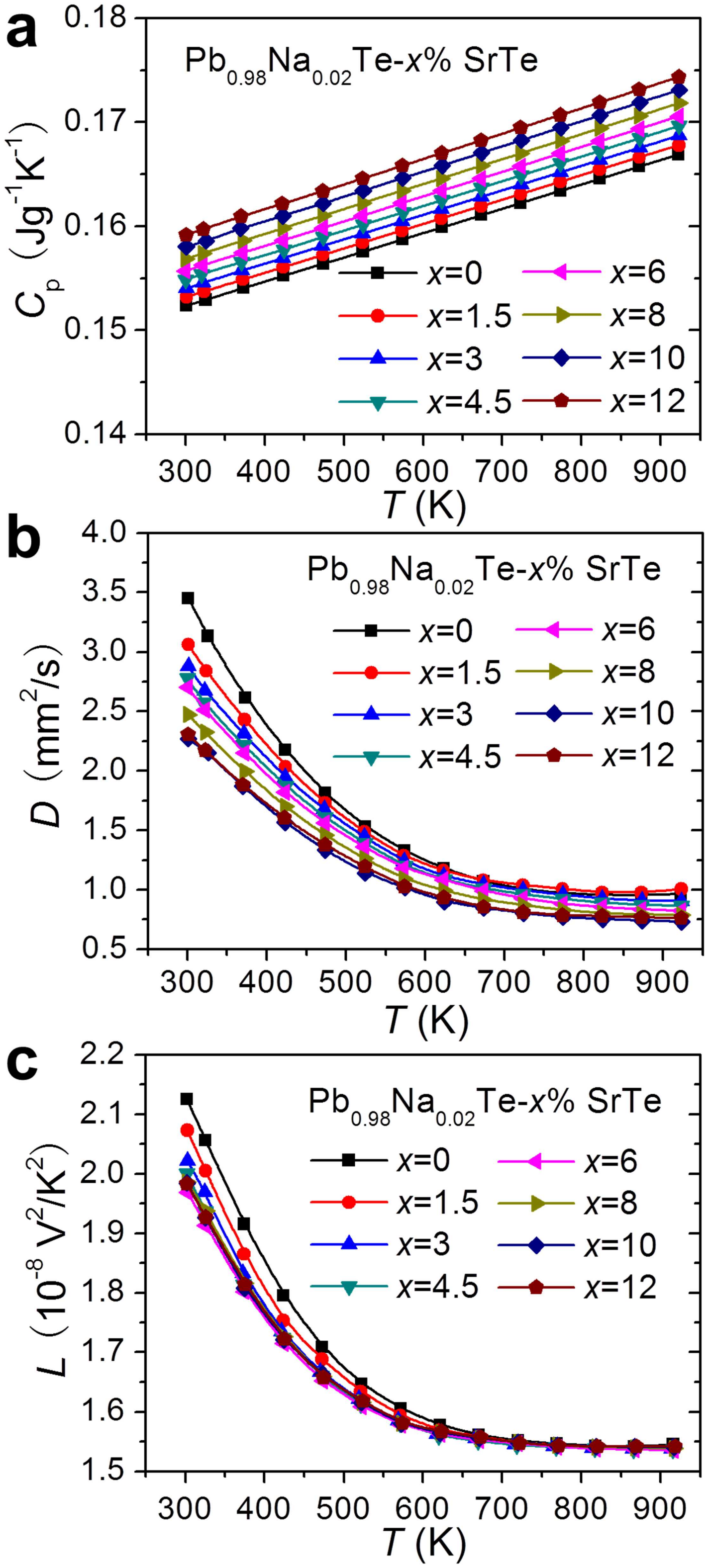

Supplementary Figure 20 | Thermal property related parameters for $Pb_{0.98}Na_{0.02}Te$-*x*%SrTe. (**a**) Heat capacity, (**b**) thermal diffusivity and (**c**) Lorenz number as a function of temperature for $Pb_{0.98}Na_{0.02}Te$-*x*%SrTe.

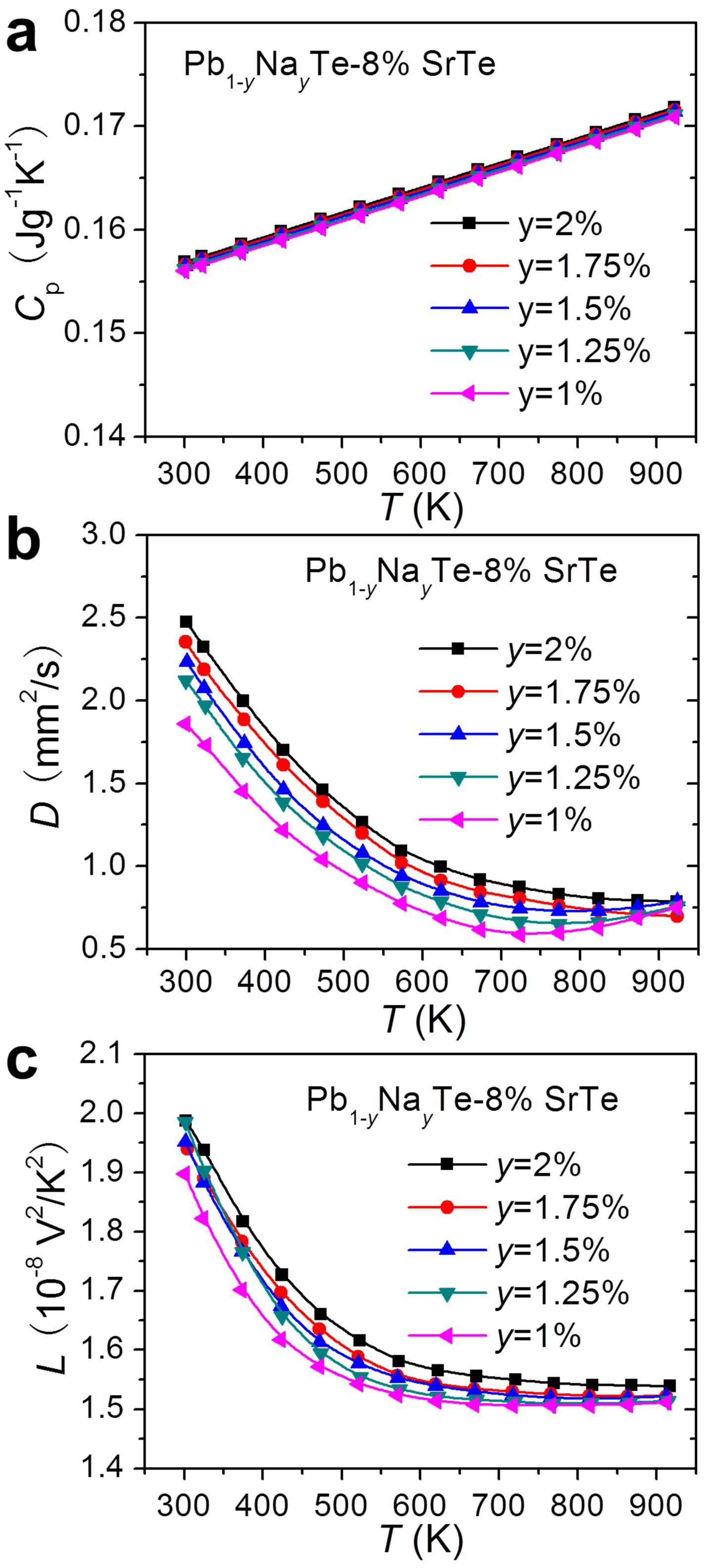

Supplementary Figure 21 | Thermal property related parameters for $Pb_{1-y}Na_yTe$-8%SrTe. (**a**) Heat capacity, (**b**) thermal diffusivity and (**c**) Lorenz number as a function of temperature for $Pb_{1-y}Na_yTe$-8%SrTe.

Supplementary Tables

Supplementary Table 1. Room temperature electrical transport properties of $Pb_{0.98}Na_{0.02}Te$-*x*%SrTe.[#]

x	± (S/cm)	*S* («V/K)	N_p (10^{20} cm^{-3})	«$_H$ (cm^2V^{-1} s^{-1})	m^* (m_e)
0	2487	62	1.37	114	0.82
1.5	2140	72	1.45	92	0.99
3	2096	80	1.67	79	1.21
4.5	2086	87	1.59	82	1.27
6	2078	93	1.69	77	1.42
8	2041	91	1.66	77	1.37
10	1868	91	1.60	73	1.34
12	1942	92	1.46	83	1.27

[#]±, electrical conductivity; *S*, Seebeck coefficient; N_p, Hall carrier density; «$_H$, Hall mobility; m^* (m_e), the effective mass of carriers.

Supplementary Table 2. Input parameters for calculation of relaxation time of phonon scattering for PbTe –8 mol% SrTe.

Parameter	Symbol (unit)	Value
Grüneisen parameter of PbTe, ref. 12	γ	1.96
Average sound velocity of PbTe, ref. 12	^{3} (m/s)	1770
Debye temperature of PbTe, ref. 12	θ_D (K)	136
Average mass of an atom of PbTe	M (kg)	2.7767×10^{-25}
Ratio of normal phonon scattering to Umklapp scattering	β	2.5
Volume per atom for PbTe	V_0 (Å^{-3})	33.69
Longitudinal phonon velocity of PbTe, ref. 12	$^{3}{}_{L}$ (m/s)	3590
Transverse phonon velocity of PbTe, ref. 12	$^{3}{}_{T}$ (m/s)	1260
Average radius of nanoscale precipitates	R (nm)	2.5
Mass density of PbTe	D_{PbTe} (kg/m^3)	8164
Mass density of SrTe	D_{SrTe} (kg/m^3)	7550
Misfit between the matrix and the precipitate	ε_0	0.031
Mass of an atom Pb	M_{Pb} (amu)	207.2
Mass of an atom Ba	M_{Sr} (amu)	87.62
Radius of an atom Pb	r_{Pb} (pm)	175
Radius of an atom Sr	r_{Sr} (pm)	200
Fractional occupant	f_{Pb}	0.9509
	f_{Sr}	0.0491
Number density of nanoscale precipitates	Vp (m^{-3})	$5.099*10^{22}$

Supplementary Table 3. Room temperature densities of $Pb_{0.98}Na_{0.02}Te$-x%SrTe and $Pb_{1-y}Na_yTe$-8%SrTe.

x	0	1.5	3	4.5	6	8	10	12
d	7.968	8.010	7.931	7.941	7.976	7.903	7.993	7.945
d_T	8.172	8.165	8.159	8.153	8.147	8.139	8.131	8.123
d%	97.5	98.1	97.2	97.4	97.9	97.1	98.3	97.8
y	1.75%		1.5%		1.25%		1%	
d	7.944		7.978		7.931		8.021	
d_T	8.148		8.157		8.167		8.176	
d%	97.5		97.8		97.1		98.1	

*The theoretical densities d_T of the $Pb_{0.98}Na_{0.02}Te$-x%SrTe and $Pb_{1-y}Na_yTe$-8%SrTe samples were calculated using the law of mixtures:

$$d(t) = \sum_{i=1}^{n} t_i A_i\mathrm{Te} \qquad \text{(Supplementary Equation 1)}$$

In Equation S1, A_i represents the cationic element, n is total number of the cationic elements, t_i is the volume fraction of the binary telluride formed between the ith cationic and Te. The following theoretical densities for binary tellurides (all in the form of cubic crystal) were used in the calculation: 8.25 g/cm^3 for PbTe (JCPDS Card #38-1435), 2.93 g/cm^3 for Na_2Te (JCPDS Card #47-1700) and 7.55 g/cm^3 for SrTe (JCPDS Card #65-0886).

Supplementary Methods

Debye-Callaway Model simulation of lattice thermal conductivity

To understand the mechanism of the phonon scattering in the PbTe-SrTe system, we carried out simulation of lattice thermal conductivity ©_{lat} based on the Debye-Callaway model[13].

$$\text{©}_{\mathrm{lat}} = \frac{k_{\mathrm{B}}}{2\text{®}^{2}c}\left(\frac{k_{\mathrm{B}}T}{\hbar}\right)^{3}\int_{0}^{\text{¯}_{\mathrm{D}}/T} \text{²}_{\mathrm{C}} \frac{e^{x}}{\left(e^{x}-1\right)^{2}} x^{4}dx \qquad \text{(Supplementary Equation 2)}$$

Where x is defined as $\hbar\omega/k_{\mathrm{B}}T$, k_{B} is the Boltzmann's constant, $\hbar$ is Plank constant, c is an average phonon-group velocity, T is absolute temperature and ¯_{D} is Debye temperature. The τ_{c} is related the scattering from Umklapp process, normal processes, nanoscale precipitates, strains, and point defects, respectively by the equation:

$$\text{²}_{\mathrm{c}}^{-1} = \text{²}_{\mathrm{U}}^{-1} + \text{²}_{\mathrm{N}}^{-1} + \text{²}_{\mathrm{P}}^{-1} + \text{²}_{\mathrm{S}}^{-1} + \text{²}_{\mathrm{PD}}^{-1} \qquad \text{(Supplementary Equation 3)}$$

The expressions of relaxation time for Umklapps process (²_{U}), normal process (²_{N}), nanoscale precipitates (²_{P}), strains (²_{S}) and point defects (²_{PD}) are adapted from Lo *et al.*[14] and He *et al.*[15]

Based on the expressions of relaxation times, we show the different contributions to phonon scattering for each mechanism in Supplementary Fig. 10. The contribution from strain induced by point defects is significant compared to the precipitates at lower frequency.

Supplementary References

1 Partin, D. L., Thrush, C. M. & Clemens, B. M. Lead strontium telluride and lead barium telluride grown by molecular‐beam epitaxy. *J. Vac. Sci. Technol. B* **5**, 686-689 (1987).

2 Beyer, H. *et al.* in *Eighteenth International Conference on Thermoelectrics.* 687-695 (IEEE, *1999*).

3 Zhao, L. D. *et al.* All-scale hierarchical thermoelectrics: MgTe in PbTe facilitates valence band convergence and suppresses bipolar thermal transport for high performance. *Energy Environ. Sci.* **6**, 3346-3355 (2013).

4 Rowe, D. M. *CRC handbook of thermoelectrics*. (CRC press, 1995).

5 Pei, Y. *et al.* Stabilizing the Optimal Carrier Concentration for High Thermoelectric Efficiency. *Adv. Mater.* **23**, 5674-5678 (2011).

6 Pei, Y., Wang, H., Gibbs, Z. M., LaLonde, A. D. & Snyder, G. J. Thermopower enhancement in $Pb_{1-x}Mn_xTe$ alloys and its effect on thermoelectric efficiency. *NPG Asia Mater.* **4**, e28 (2012).

7 Smirnov, I. K. & Ukhanov, Y. I. Investigation of the valence band of lead telluride using infrared reflection data. *Sov. Phys. Semiconductors* **3**, 1553-1556 (1970).

8 Sysoeva, L. M., Vinogradova, M. N., Kolomoets, N. V. & Ravich, Y. I. Influence of the second valence band on the thermoelectric figure of merit of p-type material. *Sov. Phys. Semiconductors* **3**, 975-977 (1970).

9 Biswas, K. *et al.* High-performance bulk thermoelectrics with all-scale hierarchical architectures. *Nature* **489**, 414-418 (2012).

10 Zhao, J. *et al.* Spectroscopic evidence for the convergence of lower and upper valence bands of PbQ (Q= Te, Se, S) with rising temperature. *arXiv preprint arXiv:1404.1807* (2014).

11 Gibbs, Z. M. *et al.* Temperature dependent band gap in PbX (X= S, Se, Te). *Appl. Phys. Lett.* **103**, 262109 (2013).

12 Zhang, Y., Ke, X., Chen, C., Yang, J. & Kent, P. Thermodynamic properties of PbTe, PbSe, and PbS: First-principles study. *Phys. Rev. B* **80**, 024304 (2009).

13 Callaway, J. Model for lattice thermal conductivity at low temperatures. *Phys. Rev.* **113**, 1046 (1959).

14 Lo, S. H., He, J., Biswas, K., Kanatzidis, M. G. & Dravid, V. P. Phonon Scattering and Thermal Conductivity in p‐Type Nanostructured PbTe‐BaTe Bulk Thermoelectric Materials. *Adv. Funct. Mater.* **22**, 5175-5184 (2012).

15 He, J., Girard, S. N., Kanatzidis, M. G. & Dravid, V. P. Microstructure‐Lattice Thermal Conductivity Correlation in Nanostructured $PbTe_{0.7}S_{0.3}$ Thermoelectric Materials. *Adv. Funct. Mater.* **20**, 764-772 (2010).
